# Molecular and Functional Characterization of Black Soldier Fly Derived Peptide Against Food-Relevant Bacteria

**DOI:** 10.3390/foods15173107

**Published:** 2026-09-01

**Authors:** Muhammad Raheel Tariq, Yanwen Liang, Hui Wang, Haiwen Lin, Chaozhong Zheng, Xiaotian Tang, Youming Liu, Jianguang Qin, Gang Lu, Fei Wang

**Affiliations:** 1 College of Food Science and Technology, Huazhong Agricultural University, Wuhan 430070, China; raheeltariq916@gmail.com (M.R.T.); lym@mail.hzau.edu.cn (Y.L.); 2 Agricultural Products Processing Research Institute, Chinese Academy of Tropical Agricultural Sciences, Zhanjiang 524001, Chinawhqz1985@163.com (H.W.);; 3 Institute of Insect Sciences, College of Agriculture and Biotechnology, Zhejiang University, Hangzhou 310030, China; tangxt@zju.edu.cn; 4 Southern Marine Science and Engineering Guangdong Laboratory (Guangzhou), Guangzhou 511458, China; jian.qin@flinders.edu.au; 5 College of Science and Engineering, Flinders University, Adelaide, SA 5001, Australia; 6 Yu Ren Tang Biotechnology (Zhanjiang) Co., Ltd., Zhanjiang 524001, China

**Keywords:** *BSF*, antimicrobial peptide, *Hermetia illucens*, natural food preservatives, foodborne pathogens

## Abstract

Microbial contamination remains a major challenge in food systems, creating demand for stable natural antimicrobials. This study investigates the inducible antibacterial response of *Hermetia illucens* and characterized the black soldier fly defensin-like peptide DLP4. Larvae were challenged with *Staphylococcus aureus* or *Escherichia coli*, and hemolymph activity, antimicrobial-peptide gene expression, C18 solid-phase extraction fractions, and LC–MS/MS profiles were evaluated. Synthetic mature DLP4 was further assessed for antibacterial spectrum, physicochemical stability, and structural properties. Hemolymph antibacterial activity varied with treatment and time, while DLP4 and CLP1 showed distinct transcriptional responses. Antibacterial activity was concentrated in early fractions, with F3 showing the greatest activity. LC–MS/MS detected a conserved DLP-family motif and a cysteine-rich defensin-like peptide in F3, whereas the complete 40-amino-acid sequence of synthetic DLP4 was confirmed. DLP4 showed strong, strain-dependent activity against Gram-positive bacteria, including *Bacillus subtilis*, *Listeria ivanovii*, and *Staphylococcus* spp., while no MIC endpoint was detected for the tested *E. coli* strains. DLP4 retained substantial activity after heat, ultraviolet, storage, pH, NaCl, and most metal-ion treatments, although Fe^3+^ reduced activity. These findings support DLP4 as a stable, Gram-positive-active antimicrobial candidate for further evaluation in food systems.

## 1. Introduction

Microbial contamination is a major challenge across the agricultural and food supply chain. It contributes to foodborne illness, product spoilage, economic losses, and shorter shelf life. Several preservation methods are used to control microbial growth, including thermal processing, refrigeration, acidification, high-pressure processing, and chemical preservatives. However, intensive treatments may adversely affect sensory properties, nutritional quality, and consumer acceptance. These concerns are particularly relevant for minimally processed foods [1,2,3,4,5]. Antimicrobial peptides are of particular interest because they can show strong antibacterial activity at low concentrations. They also exhibit considerable structural and functional diversity [4,6,7,8,9]. However, growth-inhibition data alone are not sufficient to establish the relevance of a newly identified peptide to agricultural and food applications. Its molecular identity, antibacterial spectrum, structure–function relationship, and stability under processing and formulation conditions must also be evaluated. Ultimately, practical food-system applicability also requires validation in representative food matrices together with assessment of safety, formulation, sensory effects, and processing compatibility.

Antimicrobial peptides are evolutionarily conserved components of innate immunity in microorganisms, plants, invertebrates, and vertebrates [8,9,10]. Many AMPs are short, cationic, and amphipathic. Their activity depends on the distribution of positively charged and hydrophobic residues, as well as their secondary structure and conformational flexibility. In cysteine-rich peptides, disulfide-bond organization also plays an important role [6,8,9,10]. Electrostatic attraction allows cationic peptides to bind to negatively charged microbial surfaces. This interaction promotes peptide accumulation at the cell envelope. The peptides may then disrupt the membrane, interfere with the cell wall, or act on intracellular targets [7,11,12]. Antibacterial mechanisms and target selectivity vary widely among peptide families. They cannot be predicted reliably from net charge or antimicrobial prediction tools alone. Therefore, sequence characteristics, structural behaviour, and strain-specific antibacterial activity should be evaluated together to better understand peptide function [12].

Physicochemical conditions are important when evaluating antimicrobial peptides for food-related applications. Changes in pH can affect peptide ionization, solubility, and interactions with bacterial membranes. They can also alter the surface charge of target bacteria. Temperature, ultraviolet exposure, and storage conditions may further influence peptide stability and conformation. Mono- and multivalent ions can also affect peptide aggregation, oxidation, diffusion, and interactions with microbial surfaces [8,13,14,15]. These factors can either preserve or reduce antibacterial activity. For example, the activity of nisin and other food-relevant antimicrobial peptides is strongly affected by pH, temperature, ionic conditions, and interactions with food components [2,13,14,15]. Evaluating antimicrobial peptides under defined physicochemical conditions is essential for determining their operational stability. It also helps identify the conditions under which antibacterial activity is retained. This is particularly important for cysteine-rich peptides. Their intramolecular disulfide bonds can stabilize peptide structure and may increase resistance to environmental stress [15,16,17,18,19].

Insects represent a diverse source of antimicrobial peptides because they rely strongly on innate immune responses to survive continuous exposure to microorganisms [15,19,20]. Following microbial recognition, signalling pathways induce the production of defensins, cecropins, attacins, diptericins, and other antimicrobial effectors that enter the hemolymph or are expressed at epithelial barriers [21,22,23,24,25]. Insect defensins generally contain approximately 40 amino acid residues and six conserved cysteines that support a compact cysteine-stabilized α/β architecture [16,20,26]. Many insect defensins show stronger activity against Gram-positive bacteria, although their antibacterial spectrum varies. Their activity is linked to cationic surface regions, structured loops and β-strands, and interactions with the bacterial membrane or cell wall. The integrity of the cysteine-stabilized structure is also important for maintaining their function [16,27,28,29]. Defensin-like peptides from fungi, such as plectasin and copsin, also show strong activity against Gram-positive bacteria. Their antibacterial effects are linked to specific interactions with bacterial cell-wall components. These examples highlight the importance of compact cysteine-rich structures in determining antibacterial selectivity [17,18,30].

The black soldier fly (BSF), *Hermetia illucens*, is increasingly studied in agricultural and food biotechnology. Its larvae can convert organic side streams into biomass rich in protein, lipids, and chitin [31]. Genomic and transcriptomic studies have revealed a diverse repertoire of antimicrobial peptide genes in BSF. These include defensin-like, cecropin-like, attacin-like, and diptericin-like families [32,33]. This diversity may reflect the adaptation of BSF to microbially complex substrates. It also makes BSF a valuable system for antimicrobial peptide discovery. AMP expression in BSF is influenced by diet, developmental stage, tissue type, and microbial exposure. This indicates that peptide production is regulated rather than constant [32]. However, transcriptional induction alone does not confirm that the mature peptide is present in an active biological fraction. The defined peptide sequence must be functionally tested to determine its antibacterial potency and target selectivity.

Defensin-like peptide 4 (DLP4) is encoded by a reported BSF precursor sequence deposited under GenBank accession KF805350.1. Processing of this precursor is expected to produce a short, cysteine-rich, and strongly cationic mature peptide. These features are consistent with those of insect defensins and suggest potential antibacterial activity. However, sequence classification and structural prediction alone cannot confirm the biological presence, molecular form, antibacterial spectrum, or stability of DLP4. The relationship between bacterial challenge, hemolymph antibacterial activity, DLP4 gene expression, peptide detection, and the activity of mature DLP4 also remains unclear. In addition, its stability under different pH values and other physicochemical conditions relevant to food processing and formulation has not been fully characterized. Addressing these gaps is essential to establish DLP4 as a molecularly defined antimicrobial candidate rather than solely a predicted immune effector.

Accordingly, this study investigated the antibacterial response of BSF larvae following bacterial challenge and provided an integrated molecular and functional characterization of DLP4. The study combined bacterial challenge, hemolymph antibacterial activity, AMP gene expression, bioassay-guided fractionation, and LC–MS/MS profiling within a single experimental framework. Untreated, mechanical-injury, and injection controls were included to distinguish bacterial effects from responses caused by experimental manipulation. Hemolymph antibacterial activity and AMP transcription were evaluated during the viable post-challenge period. LC–MS/MS was then used to examine the presence of DLP-family peptides in the active hemolymph fraction. Synthetic DLP4 was subsequently tested for strain-dependent antibacterial activity and stability under different pH, temperature, ultraviolet, storage, and metal-ion conditions. Sequence-based physicochemical analysis, structural modelling, membrane-positioning analysis, and circular dichroism spectroscopy were further integrated with the experimental data to investigate molecular features associated with DLP4 activity and stability.

## 2. Materials and Methods

### 2.1. Insect Rearing and Experimental Design

After hatching, BSF larvae were reared on wheat bran for the first three days. They were then randomly assigned to experimental groups and transferred to a temperature-controlled room. Larvae were maintained on wheat bran until 8 days of age [34]. To reduce developmental and dietary variation, larval age, diet composition, and rearing density were kept consistent across all groups. Rearing conditions were maintained at 27 ± 2 °C, 70% relative humidity, and a 12 h light:12 h dark photoperiod [35]. Larvae were housed in plastic containers with an internal volume of 27,000 cm^3^ (45.7 cm × 30 cm × 20 cm). Each container received 2.5 kg of wheat bran every 4 days to ensure consistent substrate availability [36]. The experiment followed a treatment × time design. Larvae were assigned to untreated, needle-pricked, and NaCl-injected control groups. Bacterial challenge groups were injected with either *S. aureus* ATCC 6538 or *E. coli* ATCC 8739 at OD_600_ values of 1.0, 2.0, 2.5, or 3.0. Intrahemocoelic injection was used to induce a systemic immune response. Injection volume, injection site, needle type, handling time, and operator technique were standardized across groups. Needle-pricked larvae served as the mechanical-injury control, whereas NaCl-injected larvae served as the vehicle control. Because injection itself can induce immune responses, bacterial effects were interpreted relative to these procedural controls. Samples were collected at 1, 2, and 3 days post-treatment. Survival was first assessed to define the suitable post-challenge period for downstream analyses. Each treatment initially contained 600 larvae (n = 600 per treatment). The same cohort was monitored for 72 h, and live and dead larvae were recorded at 24, 48, and 72 h. Survival was expressed as the percentage of larvae remaining alive relative to the initial number in each group. Because the exact time of death between observations was unknown, deaths were assigned to the first observation point at which larvae were recorded as dead. Larvae that remained alive at 72 h were right-censored. Kaplan–Meier survival curves were generated for untreated, procedural-control, and bacterial-challenge groups. Differences among survival distributions were evaluated using the log-rank (Mantel–Cox) test [20,21,22].

### 2.2. Bacterial Strains, Culture Conditions, and Dose Standardization

*S. aureus* ATCC 6538 was cultured in tryptic soy broth, while *E. coli* ATCC 8739 was cultured in Luria–Bertani broth. Fresh overnight cultures were prepared at 37 °C with shaking at 120 rpm. Cells were collected by centrifugation and washed three times with sterile physiological saline. The pellets were then resuspended in sterile 0.9% NaCl and adjusted to OD_600_ values of 1.0, 2.0, 2.5, or 3.0. Because OD_600_ does not directly represent viable cell number and may differ between bacterial species, each suspension was quantified by viable plate counting before injection. Ten-fold serial dilutions were prepared in sterile saline. Appropriate dilutions were plated on tryptic soy agar for *S. aureus* and LB agar for *E. coli*. Plates were incubated at 37 °C for 18–24 h. Colonies from countable plates were used to calculate CFU/mL. The viable dose delivered per larva was estimated by multiplying the measured CFU/mL by the injection volume of 0.005 mL. Bacterial challenge levels were therefore reported as both OD_600_ values and estimated CFU per larva [37,38]. Corresponding CFU/mL values are provided in Table S1. Before treatment, larvae were rinsed with sterile diluent and briefly surface-sterilized with 70% ethanol. They were then air-dried and immobilized on ice. For bacterial challenge, 5 µL of the bacterial suspension was injected into the hemocoel through the posterior intersegmental membrane using a Hamilton microliter syringe fitted with a fine needle. Needle-pricked controls underwent cuticle penetration without fluid injection. NaCl controls received 5 µL of sterile physiological saline. Untreated larvae were maintained under the same experimental conditions [39].

### 2.3. Hemolymph Collection and Processing

Hemolymph was collected at 24, 48, and 72 h post-treatment, corresponding to Days 1, 2, and 3. Collection was performed under cold conditions to reduce prophenoloxidase activation, melanization, and proteolysis [40]. For each treatment × time-point combination, three independent biological pools were prepared. Each pool contained hemolymph from approximately 1000 larvae and yielded about 5 mL. Thus, approximately 3000 larvae were used per treatment × time point. Each pool was processed independently and treated as one biological replicate in downstream hemolymph assays. To minimize melanization, all collection steps were performed on an ice-cold surface or in an ice bath. Hemolymph was collected directly into pre-chilled 5 mL centrifuge tubes containing phenylthiourea (PTU; final concentration 1 µg/mL) and protease inhibitor (final concentration 10 µg/mL) [36]. PTU was used to inhibit phenoloxidase activity during sample handling. After collection, tubes were kept on ice for approximately 30 s to further reduce melanization [41]. Larvae were handled aseptically, and the cephalic region was removed using sterile scissors. Hemolymph was expelled by gently pressing the remaining body into the chilled tubes. Samples were processed immediately after collection. Hemolymph was clarified by centrifugation at 12,000× *g* for 35 min at 4 °C. The cell-free supernatant was transferred to new pre-chilled tubes without disturbing the pellet. Samples were then aliquoted and stored at −80 °C until analysis. Each aliquot was thawed once on ice and used immediately to avoid activity loss caused by repeated freeze–thaw cycles [42]. For antibacterial assays, each biological pool was tested separately. Technical replicate discs or plates from the same pool were averaged before statistical analysis. When a concentrated crude antimicrobial extract was required, clarified hemolymph was lyophilized at −50 °C. The resulting powder was stored at −20 °C in sealed tubes until reconstitution and analysis.

### 2.4. Hemolymph Antibacterial Activity Assay

Hemolymph antibacterial activity was evaluated using an agar disk diffusion assay and expressed as the zone of inhibition (ZOI) [43]. *S. aureus* and *E. coli* were used as Gram-positive and Gram-negative indicator bacteria, respectively. *S. aureus* was cultured on tryptic soy agar (TSA), whereas *E. coli* was cultured on LB agar (LBA). Fresh bacterial suspensions were adjusted to 0.5 McFarland before inoculation to standardize cell density [44]. A 0.5 McFarland suspension corresponds approximately to 1.5 × 10^8^ CFU/mL for *E. coli*, although the exact viable count may vary with bacterial species and culture conditions [45]. The standardized suspensions were evenly spread over the agar surface using sterile cotton swabs to produce confluent bacterial lawns. Plates were briefly dried under aseptic conditions before disc placement. Sterile 6-mm paper discs were placed on the inoculated agar using sterile forceps. Clarified hemolymph was loaded onto each disc at a fixed volume of 5 µL [46]. Plates were incubated at 37 °C for 24 h, which fell within commonly used incubation windows for diffusion based growth inhibition readouts [44]. Plates were incubated at 37 °C for 24 h. After incubation, inhibition zones were measured across the center of each disc at the boundary of complete growth inhibition. ZOI values for hemolymph assays were recorded in centimeters, whereas those for SPE fractions were recorded in millimeters. For each treatment × day × indicator bacterium combination, the three independent hemolymph pools were tested separately. Multiple technical replicate discs were prepared from each pool. Technical replicates were averaged to obtain one value for each biological pool. These pool means were then used for statistical analysis. Thus, the biological replicate number was n = 3 independent pools per treatment × day. The assay was also used as a screening step to identify groups for further analysis. Groups showing comparatively strong antibacterial activity, particularly against *S. aureus*, were prioritized for subsequent experiments. Sterile NaCl was used as the negative control.

### 2.5. AMP Gene Expression

RT-qPCR was performed on day-1 groups of OD_600_ 2.5 to provide transcriptional context for interpretation. Total RNA was extracted from larval samples using the SteadyPure Universal RNA Extraction Kit II (Accurate Biotechnology, AG21022, Changsha, China) according to the manufacturer’s instructions. RNA was eluted in RNase-free water, handled on ice, and stored at −80 °C until reverse transcription. Residual genomic DNA was removed and first strand cDNA was synthesized using the Evo M-MLV RT Mix Kit with gDNA Clean for qPCR (Accurate Biotechnology, AG11728). Quantitative PCR was performed using SYBR Green Premix Pro Taq HS qPCR Kit II (Accurate Biotechnology, AG11741) with gene specific primers targeting DLP4, and CLP1. Primer specificity was verified by melt curve analysis and by the absence of amplification in negative controls. Relative expression was calculated using the comparative Ct method after normalization to the selected reference gene *Actin* [36,47]. The markers used in this study are listed in Table 1. Actin Ct values did not differ significantly among the untreated, needle-pricked, NaCl-injected, *S. aureus*-, and *E. coli*-challenged groups (one-way ANOVA, *p* = 0.479), supporting its use as the reference gene under the present experimental conditions (Table S2). This was also used as a screening method such as groups with higher dlp4 expression was used for further analysis.

### 2.6. Bioassay-Guided SPE Fractionation

To localize antibacterial activity biochemically, clarified hemolymph from the selected induction condition (*S. aureus* OD_600_ 2.5, Day 1) was subjected to C18 solid-phase extraction. Three independent clarified hemolymph pools were prepared for this condition, each representing an independent biological replicate. Hemolymph from each biological replicate was processed through the C18-SPE workflow under the same conditions. Samples were acidified with 0.05% trifluoroacetic acid (TFA) and loaded onto Sep-Pak Vac C18 cartridges (3 cc, 200 mg). Cartridges were conditioned with methanol and equilibrated with 0.05% TFA in water before sample loading. After washing with 0.05% TFA in water, retained material was eluted stepwise with 10–100% acetonitrile in 0.05% TFA water to generate fractions F1–F10. Each fraction was collected as a 20 mL eluate, lyophilized, reconstituted to the same final volume, and screened against *S. aureus* and *E. coli* using the disk-diffusion assay described above. Corresponding fractions obtained from the three independent biological preparations were assayed separately. Technical replicate disks within each biological replicate were averaged before analysis. Thus, n = 3 represents three independent biological replicates for each F1–F10 fraction [48].

### 2.7. SDS-PAGE of Active Fraction

Protein concentration of purified F3 was determined via enhanced BCA assay (Pierce) using bovine serum albumin as a standard and monitored at 562 nm. SDS–PAGE was performed to assess the protein composition of the most active SPE fraction, F3 20 µg, using the discontinuous Laemmli Tris–glycine buffer system. Samples were prepared in Laemmli sample buffer under reducing conditions, heat-denatured at 96 °C for 5 min, and loaded onto 4–20% gradient SDS–PAGE gels. Electrophoresis was carried out in Tris–glycine–SDS running buffer at constant voltage until the tracking dye reached the gel terminus. A prestained molecular-weight marker spanning 3.5–240 kDa was run in parallel to estimate apparent mass. Following electrophoresis, gels were stained with GelCode™ Blue Safe Protein Stain, destained in ultrapure water, and imaged using a gel documentation system. Apparent molecular masses were estimated by comparison with the prestained marker [49].

### 2.8. LC-MS/MS Analysis and Protein Identification

A band of low-molecular-weight of F3 was excised as previously described [50]. F3 and synthetic DLP4 samples were dissolved in 50 mM ammonium bicarbonate, reduced with 10 mM DTT at 56 °C for 1 h, alkylated with 20 mM iodoacetamide for 40 min in darkness and desalted using C18 StageTips following [51]. Peptides were separated on a C18 nano-LC column using a 66 min acetonitrile gradient and analysed by Q-Exactive mass spectrometry in Top20 data-dependent acquisition mode (Thermo Fisher Scientific Inc., Waltham, MA, USA). Full-MS and MS/MS spectra were acquired at resolutions of 70,000 and 17,500, respectively, with NCE 28 and precursor charge states of 3+–8+, following established Orbitrap-based proteomic approaches [52]. LC–MS/MS-derived peptide sequences were matched against translated BSF transcriptome contigs using SEQtools. Candidate identities were then verified against UniProtKB, NCBI Protein/RefSeq, and published BSF peptide datasets. Peptides were assigned established names only when the partial sequence and corresponding translated showed an unambiguous match; otherwise, they were conservatively classified as peptide candidates. Raw MS/MS data were processed using PEAKS Studio 10.6 (https://www.bioinfor.com/peaks-studio/, accessed on 28 June 2026) and searched against the UP000594454_343691_DLP4 database. Searches were performed using a non-specific enzyme setting, with carbamidomethylation of cysteine as a fixed modification and oxidation of methionine and protein N-terminal acetylation as variable modifications. Peptide and fragment mass tolerances were set to 10 ppm and 0.02 Da, respectively, and peptide identifications were controlled at a 1% false-discovery rate (FDR) using Q-values. LC–MS/MS-derived peptide sequences were further compared with translated BSF transcriptome contigs using SEQtools, and candidate identities were verified against UniProtKB, NCBI Protein/RefSeq, and published BSF peptide datasets. Established peptide names were assigned only when the detected sequence provided an unambiguous match; otherwise, sequences were conservatively reported as peptide candidates.

### 2.9. Determination of MIC of DLP4

DLP4 peptide was obtained from GL Biochem Co., Ltd. (Shanghai, China) used in the linear, non-oxidized form, no additional oxidative folding or directed disulfide-bond formation was performed. The analysis report of the peptide including HPLC and ESI-MS is in the Supplementary Material (Figures S9 and S10). Used for experimental characterization based on the sequence used in the gene transcription previously described in [53]. The MIC of DLP4 against *S. aureus* KCCM 40881, *S. aureus* KCCM 12256, *B. subtilis* KCCM 11316, *S. aureus* ATCC 6538, *E. coli* KCCM 11234, *E. coli* CVCC 1515, *E. coli* ATCC 8739 *L. ivanovii*, *S. hyicus*, and *S. aureus* CICC 10001 determined by broth microdilution in sterile 96-well plates. DLP4 solutions covering approximately 0.03–3.2 µM were prepared in Mueller–Hinton broth. Concentrations within this range were selected to determine the inhibitory concentration interval for each bacterial strain. Fresh logarithmic-phase cultures of each tested bacterial strain were diluted to obtain a final inoculum of approximately 5 × 10^5^ CFU/mL in each well. Wells contained equal volumes of peptide solution and bacterial suspension. Growth control, sterility control, peptide blank, and antibiotic control wells were included. Plates were incubated at 37 °C for 18–24 h. MIC was defined as the lowest DLP4 concentration showing no visible bacterial growth [54]. MIC assays were performed in three independent experiments. For each strain, the MIC was defined as the lowest peptide concentration showing no visible growth in each experiment. When replicate experiments yielded different MIC endpoints, the result was reported as the minimum–maximum range of the independently determined MIC values.

### 2.10. Functional Stability of DLP4

The functional physicochemical stability of DLP4 was assessed by measuring residual antibacterial activity after UV exposure, storage, thermal treatment, metal-salt treatment, pH treatment, and NaCl exposure, with modifications from previously reported antimicrobial peptide stability assays and standard disk-diffusion procedures. DLP4 powder (1 mg) was dissolved in 1 mL of sterile ultrapure water to obtain a concentration of 1 mg/mL. A 20 µL aliquot, corresponding to 20 µg DLP4, was used for each treatment. For UV stability, DLP4-loaded sterile paper discs were exposed to 30 W UV irradiation at room temperature for 30, 60, or 90 min. For storage stability, DLP4 was stored at 4 °C, −20 °C, or −80 °C for 1 day before antibacterial testing. For thermal stability, DLP4 aliquots were incubated at 37, 50, 60, 80, or 100 °C for 1 h, cooled to room temperature, and then tested for antibacterial activity. For metal-salt stability, DLP4 was incubated with KCl, CaCl_2_·2H_2_O, MgCl_2_, ZnCl_2_, FeCl_3_, CuSO_4_, or anhydrous CaCl_2_ at a final concentration of 5 mM at 37 °C for 3 h before antibacterial testing. For evaluation of pH stability, DLP4 solutions were adjusted to pH 2.0, 4.0, 6.0, 7.4, 9.0, or 11.0 using sterile HCl or NaOH. Samples were incubated at 37 °C for 2 h. Following exposure, all samples were readjusted to pH 7.4 before antibacterial activity was determined by the paper-disc diffusion assay. An untreated DLP4 solution maintained at pH 7.4 was used as the control. For evaluation of NaCl stability, DLP4 was incubated with NaCl at final concentrations of 3%, 4%, 5%, or 6% (*w*/*v*). Samples were incubated at 37 °C for 3 h, after which antibacterial activity was determined by the paper-disc diffusion assay without removing NaCl. DLP4 treated with an equivalent volume of sterile ultrapure water served as the control, and corresponding NaCl solutions without DLP4 were included as negative controls. All treatments were tested in triplicate, and data were expressed as mean ± standard deviation. Relative inhibition-zone diameter was calculated relative to untreated DLP4 using the equation: Relative inhibition-zone diameter (%) = (mean inhibition zone of treated DLP4/mean inhibition zone of untreated DLP4) × 100. Data were analysed in GraphPad Prism by one-way ANOVA followed by Dunnett’s multiple-comparison test against untreated DLP4, with significance set at *p* < 0.05. Sterile water and corresponding salt-only treatments were included as negative controls [36].

### 2.11. Sequence Source and Precursor-Processing Analysis

DLP4 was evaluated as an antibacterial peptide described in a previous study [48]. a stepwise sequence and structure based workflow. The precursor sequence used for all defensin-like peptide 4 precursor (DLP4) mRNA, complete cds (GenBank accession, KF805350.1). Signal peptide presence and cleavage site position were predicted using SignalP 6.0 in Eukarya mode, which is designed to identify signal peptides and their cleavage boundaries from amino-acid sequences [55]. Putative propeptide convertase cleavage sites were then evaluated using ProP v1.0b, which predicts basic residue associated processing motifs in secretory precursors [56]. The mature DLP4 sequence used for all downstream analyses was defined by the dominant combination of predicted signal peptide removal and downstream precursor processing. The mature DLP4 peptide was used for both antibacterial validation and biophysical characterization. Antibacterial validation was performed by MIC assays, whereas circular dichroism spectroscopy was used to assess solvent-dependent secondary-structure behavior. The DLP4 peptide was characterized using the ExPASy ProtParam and PeptideMass tools to determine peptide length, theoretical molecular weight, theoretical isoelectric point, charged residue composition, instability index, aliphatic index, grand average of hydropathicity (GRAVY), extinction coefficient, and expected intact masses [57]. These analyses were used to determine whether DLP4 conformed to the expected physicochemical profile of a cationic antibacterial peptide and to provide reference parameters for subsequent interpretation of mass and structure related data. To assess whether the mature DLP4 sequence was compatible with known AMP property space, antimicrobial propensity prediction was performed using AMP Scanner Vr.2 and the CAMP/CAMPR platform [58,59]. Concordant classification across these tools was considered supportive evidence that DLP4 was consistent with the sequence features of a candidate AMP. Residue level structural features of mature DLP4 were first examined using NetSurfP-3.0, including predicted secondary structure state, relative solvent accessibility, and disorder propensity [60]. These outputs were used to determine whether the mature peptide was more consistent with an ordered cysteine rich scaffold than with an intrinsically disordered sequence.

### 2.12. 3D Structural Modeling

Three-dimensional structural models of mature DLP4 were generated using two complementary prediction frameworks, ColabFold with the AlphaFold2 backend and AlphaFold Server implementing AlphaFold 3 [61,62,63]. ColabFold derived models were used to obtain standard confidence metrics, including predicted local distance difference test (pLDDT), predicted TM score (pTM), and predicted aligned error (PAE), whereas the AlphaFold Server model was used as an additional structure supported representation of the mature DLP4 fold. Structural models were examined for overall fold organization, including the presence of α-helical, β-strand, and loop regions, as well as the compactness of the predicted peptide architecture. Agreement between the ColabFold/AlphaFold2 and AlphaFold Server/AlphaFold 3 predictions was interpreted as supportive evidence for a defensin-like structural organization of DLP4.

### 2.13. Membrane-Association Analysis

Because DLP4 showed predominantly Gram-positive antibacterial activity, structure-based membrane positioning was evaluated using PPM 3.0 with a neutral DOPC bilayer and a Gram-positive bacterial plasma-membrane model. Predicted membrane orientation, insertion depth, transfer energy, and interfacial association were examined. These calculations were interpreted as computational estimates of membrane-association propensity rather than direct evidence of membrane binding or disruption [64].

### 2.14. DLP4 Circular Dichroism Analysis

Far-UV circular dichroism (CD) spectroscopy was performed using a Bio-Logic MOS-500 spectropolarimeter (Bio-Logic Science Instruments, Seyssinet-Pariset, France) to evaluate the secondary-structure behavior of DLP4 under aqueous and structure-modulating conditions. The peptide was prepared at a final concentration of 0.2 mg/mL for all CD measurements. DLP4 spectra were recorded in ultrapure water, 40 mM sodium dodecyl sulfate (SDS), 50% trifluoroethanol (TFE), 5 mM sodium acetate containing 15 mM NaCl at pH 5.0, and 5 mM Tris-HCl containing 150 mM NaCl at pH 7.4. The 40 mM SDS condition was selected as an anionic micellar membrane-mimetic environment because this concentration is above the critical micelle concentration of SDS and therefore supports micelle formation. The 50% TFE condition was included as a structure promoting solvent to evaluate the conformational tendency of DLP4 under reduced aqueous polarity. Spectra were collected at room temperature using a quartz cuvette with a 0.1 cm path length. Far-UV CD spectra were recorded over the wavelength range of 190–260 nm. For each solvent or buffer condition, a matched blank containing the same solvent or buffer without peptide was measured separately under identical instrumental settings. The corresponding blank spectrum was subtracted from each peptide spectrum before downstream processing. Blank-corrected replicate spectra were used for all analyses, whereas non-blank-subtracted spectra were excluded from interpretation. Matched blank-subtracted replicate CD spectra were averaged, converted to mean residue ellipticity, and deconvoluted over 190–240 nm using the SP175-derived DS5-4 reference basis with non-negative structural fractions constrained to sum to 100%, and fit quality was evaluated using NRMSD [65,66,67].

Raw CD signals were exported in millidegrees and converted to mean residue ellipticity (MRE) using the following equation:

$${\left[\theta \right]}_{MRE}=\frac{\theta_{mdeg}\times MRW}{10\times c\times l}$$

where $\theta_{mdeg}$ is the observed ellipticity in millidegrees, MRW is the mean residue weight, ccc is the peptide concentration in mg/mL, and *l* is the cuvette path length in cm. For DLP4, the molecular weight was 4275.08 Da, the mature peptide length was 40 amino acids, and the calculated MRW was 106.877 Da/residue. With a peptide concentration of 0.2 mg/mL and a path length of 0.1 cm, the conversion factor was 534.385. Therefore, MRE values were calculated as:

$${\left[\theta \right]}_{MRE}=\theta_{mdeg}\times 534.385$$

MRE values were expressed as deg·cm^2^·dmol^−1^. Replicate spectra were averaged after blank correction and MRE conversion. Final curves were smoothed using Savitzky–Golay smoothing with a 15 point window, polynomial order 3, and derivative order 0. Smoothed spectra were used only for visualization and comparison of spectral shape, while the converted replicate-mean values were retained as the analytical data. Conditions showing incomplete peptide dissolution or visible precipitation were excluded from spectral interpretation.

### 2.15. Statistical Analysis

All analyses were performed using GraphPad Prism 8. Survival was analyzed using Kaplan–Meier analysis appropriate to the time-specific structure of the data. For hemolymph antibacterial assays, the biological unit was the independently prepared hemolymph pool. Three independent pools were generated for each treatment × day combination, and technical replicate discs/plates from the same pool were averaged before analysis. The resulting pool means were log10(zone + 0.05)-transformed and analyzed by two-way ANOVA with treatment and day as fixed factors, followed by Dunnett’s multiple-comparisons test against the NaCl-injected control within each day when the interaction term was significant. RT-qPCR data were analyzed on day 1 using one-way ANOVA followed by Dunnett’s multiple-comparisons test with the Prick group as the reference.

## 3. Results and Discussion

### 3.1. Induction Window

**Figure 1:**
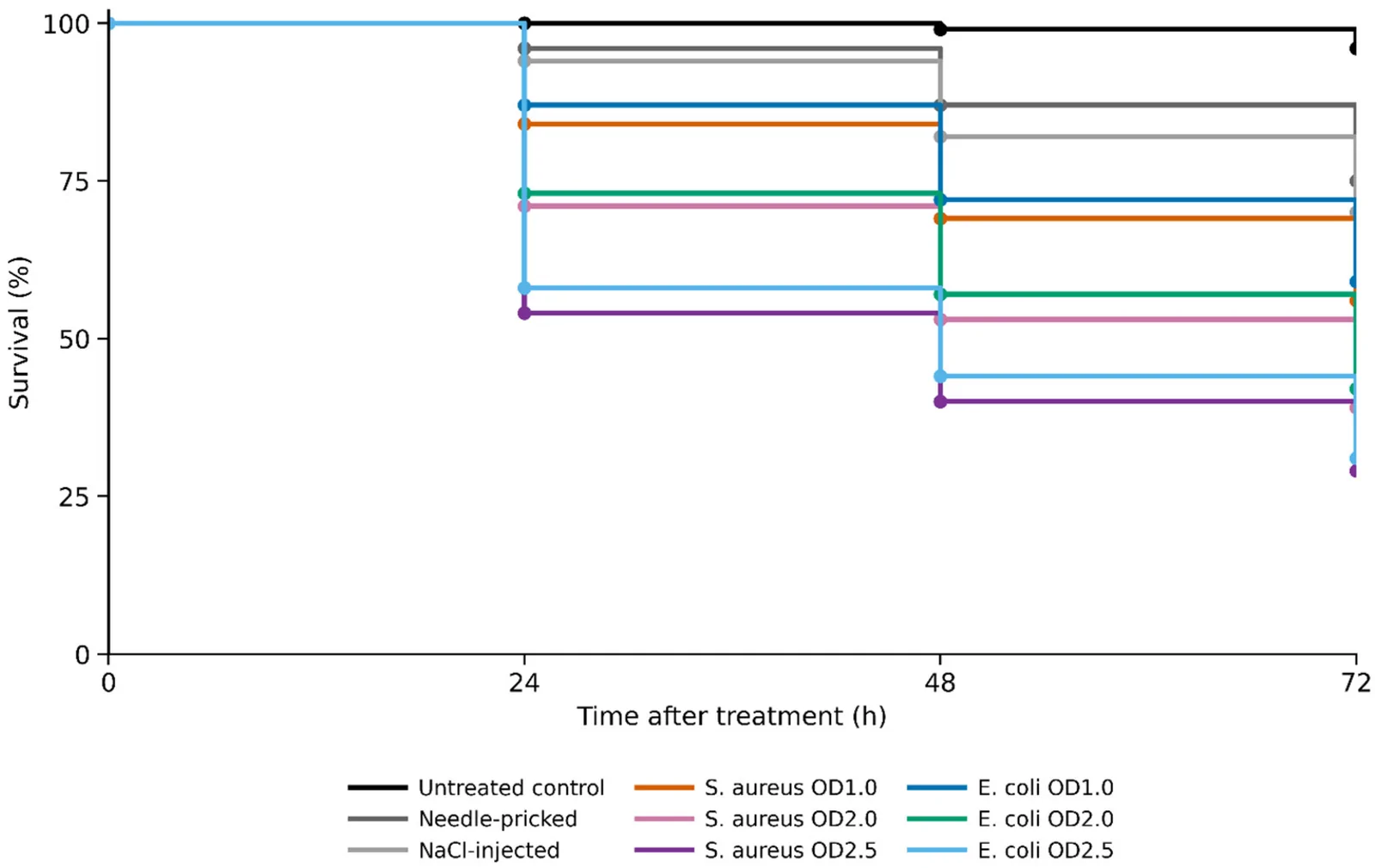
Kaplan–Meier survival analysis of BSF larvae after procedural injury and bacterial challenge. Each treatment group initially contained 600 larvae, and the same cohort was monitored at 24, 48, and 72 h after treatment. Deaths recorded at each observation interval were assigned to the corresponding time point for Kaplan–Meier analysis. Untreated, needle pricked, and NaCl injected groups were included as controls. Bacterial challenge with *S. aureus* and *E. coli* produced an OD_600_ dependent reduction in larval survival, with the strongest mortality observed in the OD_600_ 2.5 groups.

Across both bacterial species, the highest inoculum level (OD_600_ = 3.0) caused complete mortality within the 1-day post-injection monitoring window under the present rearing and injection conditions. This established OD_600_ = 3.0 as a lethal boundary for the present system rather than a dose suitable for downstream phenotypic or compositional interpretation. Accordingly, the OD_600_ = 3.0 groups were retained only for survival analysis to define the operational upper limit of tolerance, and were excluded from growth and compositional analyses because non-mortality outcomes are not meaningfully interpretable in the presence of complete mortality Figure 1.

### 3.2. Hemolymph Antibacterial Activity Against S. aureus and E. coli

**Figure 2:**
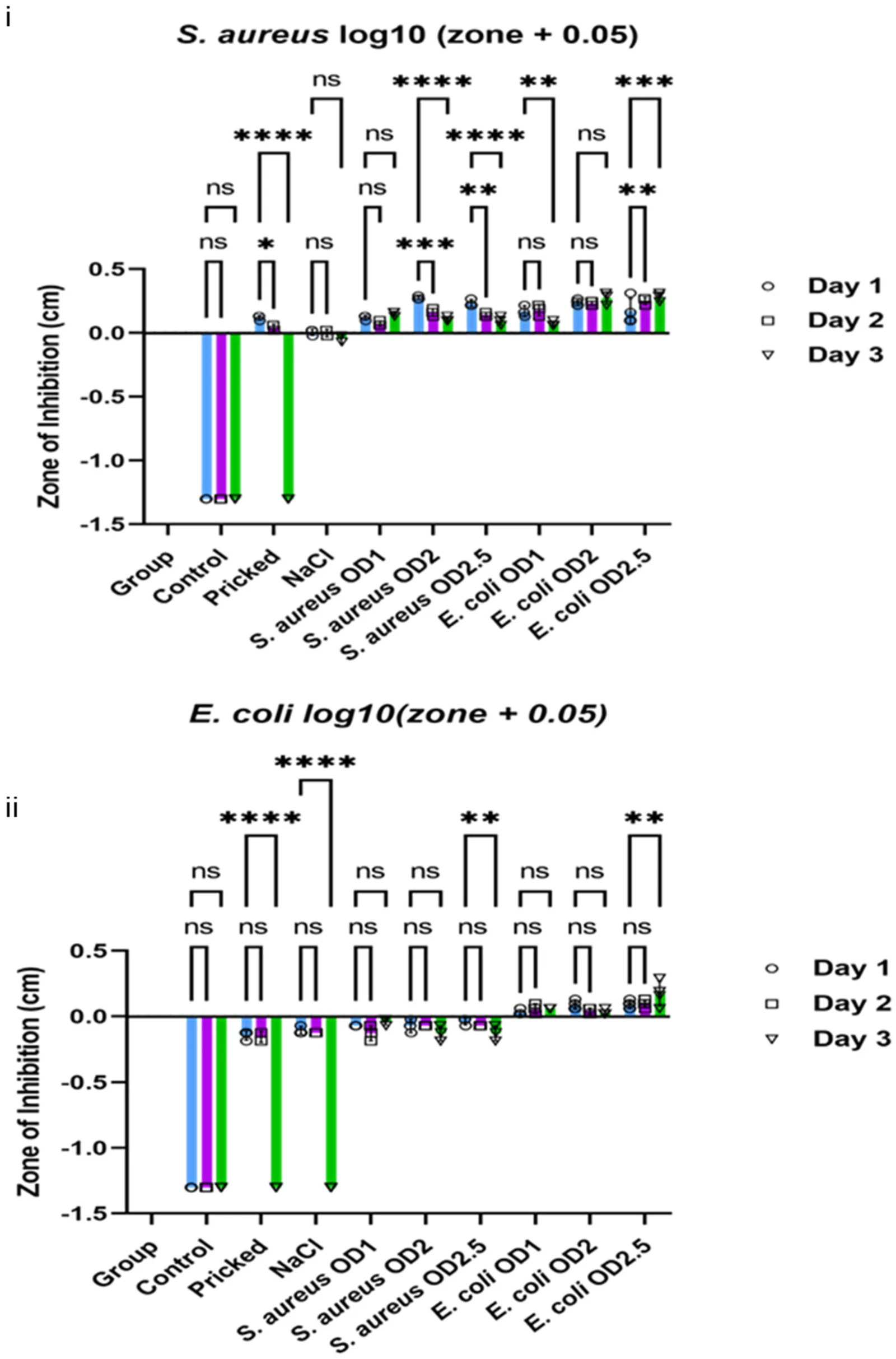
Hemolymph antibacterial activity against *S. aureus* (**i**) and *E. coli* (**ii**) at 24, 48, and 72 h post-treatment. Day 1, Day 2, and Day 3 correspond to 24, 48, and 72 h after treatment, respectively (n = 3). Bars represent mean ± SD from three independent hemolymph pools per treatment × time point, after averaging technical replicate discs within each pool. Statistical analysis was performed on log10(zone + 0.05) transformed values using two-way ANOVA followed by within-day multiple-comparison testing versus the NaCl-injected control. Sterile blank discs and NaCl-loaded discs were used as negative controls, and gentamicin was used as a positive antibacterial control Statistical comparisons are indicated by brackets. ns, not significant (*p* ≥ 0.05); * *p* < 0.05; ** *p* < 0.01; *** *p* < 0.001; and **** *p* < 0.0001.

In the *S. aureus* assay, pool mean raw inhibition zones from three independent hemolymph pools per treatment × day were analyzed by two-way ANOVA after log10(zone + 0.05) transformation. A strong Treatment × Day interaction was detected, indicating that the temporal pattern differed by treatment rather than following a uniform day trend. Because the interaction was significant, inference focused on within day comparisons against the NaCl-injected control using Dunnett-adjusted testing. These data indicate that hemolymph antibacterial activity against the Gram-positive indicator was strongly treatment dependent and time structured (Figure 2i,ii). A parallel pattern was observed in the *E. coli* assay. Pool mean raw inhibition zones from three independent hemolymph pools per treatment × day were analyzed by two-way ANOVA after log10(zone + 0.05) transformation. A significant Treatment × Day interaction again indicated that temporal change depended strongly on treatment. As in the *S. aureus* assay, inference focused on within day comparisons versus the NaCl-injected control. These findings indicate that hemolymph antibacterial activity against the Gram-negative indicator was likewise treatment dependent and time structured rather than static thus we used the Day 1 (24 h) groups for further analysis (Figures S1 and S2).

### 3.3. RT-qPCR Analysis of Immune Pathway Markers

**Figure 3:**
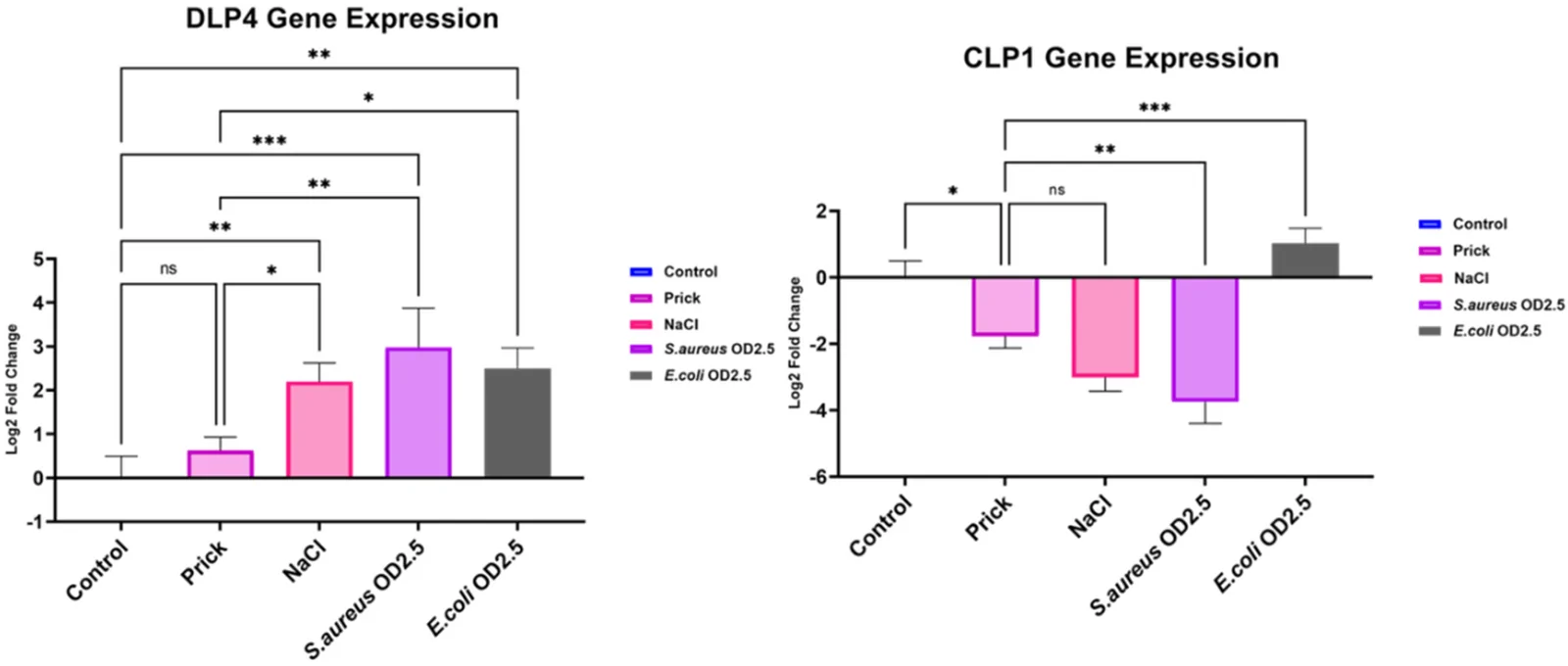
Day-1 RT–qPCR analysis of DLP4 and CLP1 transcription across procedural and bacterial challenge groups. RT–qPCR was used to quantify DLP4 transcript abundance in BSF at day 1 in the control, needle pricked, NaCl injected, *S. aureus* OD2.5, and *E. coli* OD2.5 groups. Expression is presented as log_2_ fold change [log_2_(2^−ΔΔCt^)] relative to the calibrator, with bars showing mean ± SD. Statistical testing was performed on log_2_-transformed fold-change values using one-way ANOVA followed by Dunnett’s multiple comparisons test, with Prick group used as the reference group. The horizontal baseline at 0 indicates no change relative to the calibrator, corresponding to fold change = 1. Statistical comparisons are indicated by brackets. ns, not significant (*p* ≥ 0.05); * *p* < 0.05; ** *p* < 0.01; and *** *p* < 0.001. (n = 3).

At 24 h post-treatment, DLP4 and CLP1 showed distinct transcriptional responses (Figure 3). DLP4 transcript abundance increased following NaCl injection as well as bacterial challenge. NaCl injection itself produced a significant increase in DLP4 expression compared with the untreated and needle-pricked groups, demonstrating that DLP4 induction was not specific to bacterial exposure. The bacterial challenge groups also showed elevated DLP4 expression, with the highest mean level following *S. aureus* OD_600_ 2.5 challenge, followed by *E. coli* OD_600_ 2.5. However, because the bacterial treatments were not demonstrated to be significantly different from the NaCl-injected procedural control, these results indicate a broad injection-associated DLP4 response, with bacterial challenge associated with additional numerical variation in transcript abundance.

### 3.4. SPE Fractionation

Crude hemolymph from the selected *Staphylococcus aureus* OD2.5 (24 h) induction condition was chosen because of high antibacterial activity against *S. aureus* and no activity against *E. coli* as well as high dlp4 gene expression, to be separated by stepwise C18 solid-phase extraction using a 10–100% acetonitrile (ACN) gradient in 0.05% TFA water, generating ten fractions (F1–F10) (Table 2). This fractionation scheme distributed retained components across increasing hydrophobicity and enabled subsequent bioassay-guided localization of antibacterial activity.

### 3.5. Bioassay of Active Fraction

**Figure 4:**
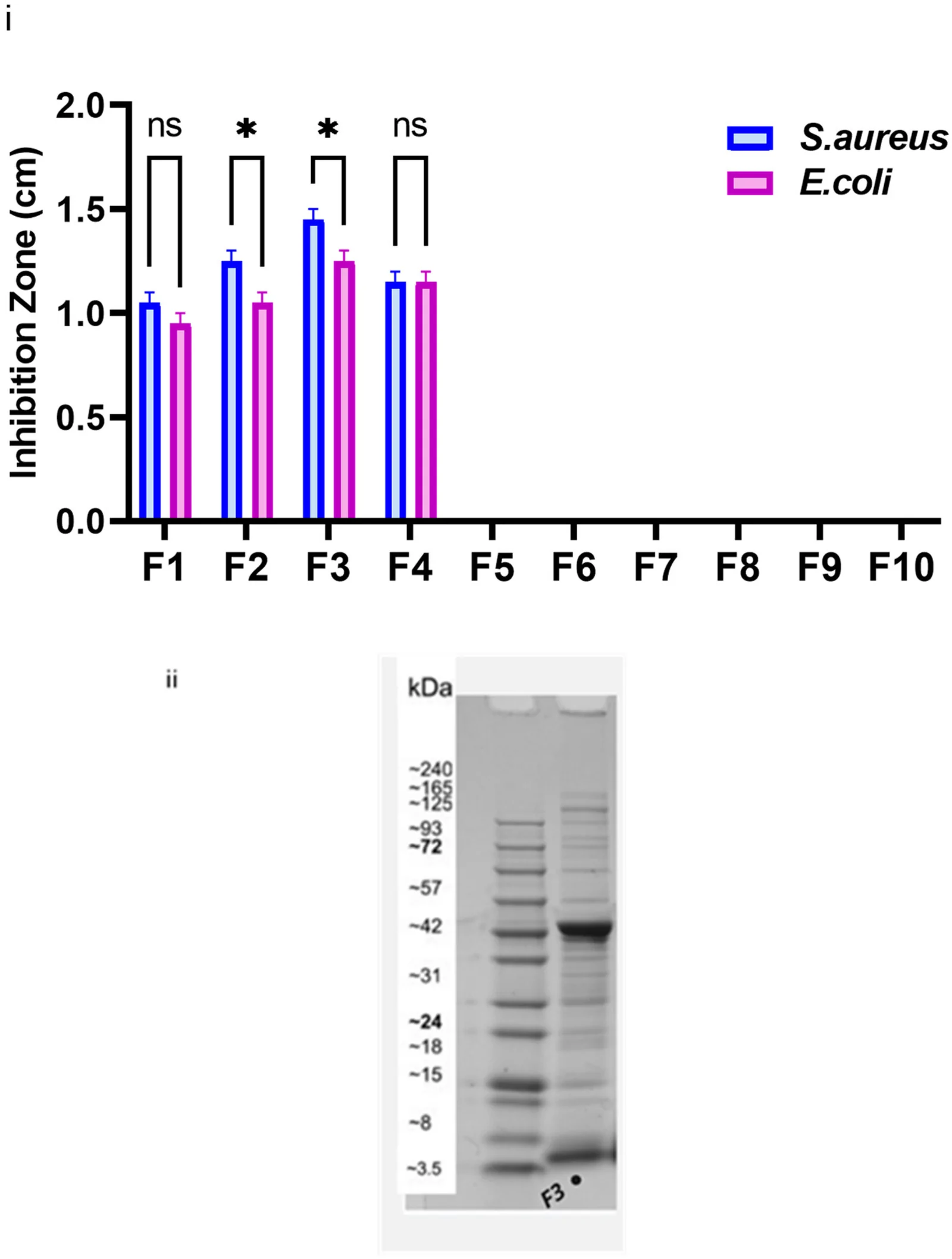
Bioassay-guided C18-SPE fractionation and SDS–PAGE analysis of the active F3 fraction. (**i**) Quantitative inhibition-zone analysis showing antibacterial activity mainly in early SPE fractions F1–F4, with F3 producing the largest zones against both indicator bacteria measured in cm. Data are presented as mean ± SD from three independent biological replicates (n = 3), with technical replicate disks averaged within each biological replicate. (**ii**) SDS–PAGE profile of fraction F3 using a 3.5–240 kDa molecular-weight marker. F3 showed a dominant low-molecular-weight band between 3.5 and 8 kDa indicating that the active fraction remained heterogeneous. Statistical comparisons are indicated by brackets. ns, not significant (*p* ≥ 0.05); * *p* < 0.05.

Bioassay-guided screening of the C18-SPE fractions showed that antibacterial activity was concentrated in the early fractions F1–F4, with F3 producing the largest inhibition zones against both indicator organisms measured in cm (Figure 4i). Activity against *S. aureus* increased from F1 to F3 and then declined in F4. Against *E. coli*, the same broad ranking was observed, but zones in the central active fractions were smaller, indicating a relatively Gram-positive-biased activity profile under the assay conditions. No measurable inhibition zones of detectable activity at the tested reconstitution volume were observed for F5–F10 (Figure S3). These data show that the antibacterial phenotype present in crude hemolymph can be narrowed to an early elution window by SPE fractionation. Because SPE fractions were lyophilized and reconstituted before testing, inhibition-zone diameters in Figure 4 should not be directly compared with crude hemolymph zones in Figure 2. Larger zones in SPE fractions may reflect concentration of active material during fraction processing.

### 3.6. LC-MS/MS Analysis

LC–MS/MS analysis of the F3 fraction detected several peptide-spectrum matches corresponding to proteins in the *Hermetia illucens* database Table 3 (Table S3) while the results are shown in chromatogram (Figure S7). The DLP-related peptide ATC(+57.02)DLL was detected with a −10lgP score of 21.18, a mass error of 1.5 ppm, an *m/z* of 692.32935 at charge state 1+, a retention time of 8.6615 min, and a peak area of 374,000. The +57.02-Da modification represented carbamidomethylation of the cysteine residue. The value of 4275.05 Da represents the average molecular mass of unmodified mature DLP4, whereas the calculated monoisotopic mass of the unmodified peptide is approximately 4272.0795 Da. Carbamidomethylation adds 57.02146 Da to each cysteine residue; modification of all six cysteines therefore adds 342.1288 Da, giving a theoretical carbamidomethylated monoisotopic mass of approximately 4614.2083 Da, in close agreement with the experimentally identified neutral mass of 4614.2082 Da. The peptide eluted at 7.9996 min and provided 100% coverage of the mature DLP4 sequence. This six-amino-acid sequence corresponded to the conserved N-terminal ATCDLL motif of the DLP1–DLP4 defensin-like peptide family. The same peptide was assigned to DLP4 and the database entries A0A7R8UAK5, A0A7R8UC55, A0A7R8YL58, and A0A7R8UB01, corresponding to HERILL_LOCUS584, HERILL_LOCUS589, HERILL_LOCUS585, and HERILL_LOCUS586, respectively. Therefore, the detection of ATCDLL supported the presence of one or more DLP-family peptides in the F3 fraction, although this shared sequence did not permit discrimination among the individual DLP1–DLP4 isoforms. It therefore provides evidence only for the presence of one or more DLP-family peptides in F3. A cysteine-rich 39-amino-acid peptide, ATCDLLSPFGVGHAACAVHCIAMGRRGGWCDDRAVCNCR, was also detected in the F3 fraction. After carbamidomethylation of its six cysteine residues, this peptide was observed at an *m/z* of 634.28412 with a charge state of 7+, a retention time of 8.4333 min, a mass error of −2.4 ppm, a −10lgP score of 15.07, and a peak area of 135,000. The sequence was assigned to the protein accessions A0A7R8UB01 and A0A7R8UC55, corresponding to HERILL_LOCUS586 and HERILL_LOCUS589, respectively. This sequence was distinct from the mature DLP4 sequence and was therefore reported separately as a database-matched cysteine-rich defensin-like peptide Table 3. Additional peptide-spectrum matches detected in the F3 fraction are presented in Table S3. Short peptides and sequences shared by multiple database proteins were interpreted cautiously because of their limited protein specificity.

LC–MS/MS analysis of the synthetic DLP4 standard provided direct sequence-level confirmation of the synthesized peptide (Supplementary Table S3) while the results are shown in chromatogram (Figure S8). The complete 40-amino-acid mature DLP4 sequence, ATCDLLSPFKVGHAACAAHCIARGKRGGWCDKRAVCNCRK, was detected with all six cysteine residues present in their carbamidomethylated forms. The full-length modified peptide produced a −10lgP score of 45.84, an experimental neutral mass of 4614.2082 Da, a mass error of 4.3 ppm, and an *m/z* of 577.78577 at charge state 8+. It eluted at 7.9996 min and produced a peak area of 10,600,000, providing 100% coverage of the mature DLP4 sequence.

In addition to the complete sequence, several nested N-terminal DLP4-derived peptides ranging from 10 to 21 amino acids were identified. The 19-amino-acid sequence ATCDLLSPFKVGHAACAAH produced the highest −10lgP score of 46.10 and provided 47.5% sequence coverage. It was detected at an *m/z* of 675.99493 with a charge state of 3+, a retention time of 8.4271 min, a mass error of 3.4 ppm, and a peak area of 107,000. Other identified sequences included ATCDLLSPFK, ATCDLLSPFKVG, ATCDLLSPFKVGH, ATCDLLSPFKVGHAA, ATCDLLSPFKVGHAAC, ATCDLLSPFKVGHAACAA, and ATCDLLSPFKVGHAACAAHCI. These nested peptides covered the N-terminal region of DLP4 and were detected with low mass errors of 0.6–1.9 ppm, further supporting the identity of the synthetic peptide.

Collectively, the detection of the complete mature sequence and multiple overlapping sequence-derived ions confirmed the identity of the synthetic DLP4 standard. In the F3 fraction, LC–MS/MS detected the conserved ATCDLL motif associated with the DLP defensin family, together with a cysteine-rich defensin-like peptide. These findings support the presence of DLP-family antimicrobial peptides in the biologically active F3 fraction.

### 3.7. MIC Assays

DLP4 exhibited clear strain-dependent antibacterial activity, with substantially greater activity against Gram-positive bacteria than against Gram-negative bacteria. Among the tested strains, *B. subtilis* KCCM 11316 was the most susceptible, with an MIC of 0.03–0.04 µM. DLP4 also inhibited the tested *S. aureus* strains. The MIC values against *S. aureus* KCCM 40881 and *S. aureus* KCCM 12256 were 0.61–1.11 and 1.15–2.12 µM, respectively. Against *S. aureus* ATCC 6538, the MIC ranged from 0.44 to 0.93 µM and *S. aureus* CICC 10001 was 1.4–1.7 µM. These results indicate that DLP4 exerted consistent inhibitory activity against the examined Gram-positive bacteria, although susceptibility varied among strains. In contrast, no MIC endpoint was detected against the Gram-negative strains *E. coli* KCCM 11234, *E. coli* CVCC 1515, or *E. coli* ATCC 8739 within the concentration range tested Table 4. Overall, the MIC profile demonstrates that DLP4 preferentially inhibits Gram-positive bacteria, particularly *B. subtilis* and *S. aureus*, while showing limited or undetectable activity against the examined *E. coli* strains.

### 3.8. Functional Physicochemical Stability of DLP4

**Figure 5:**
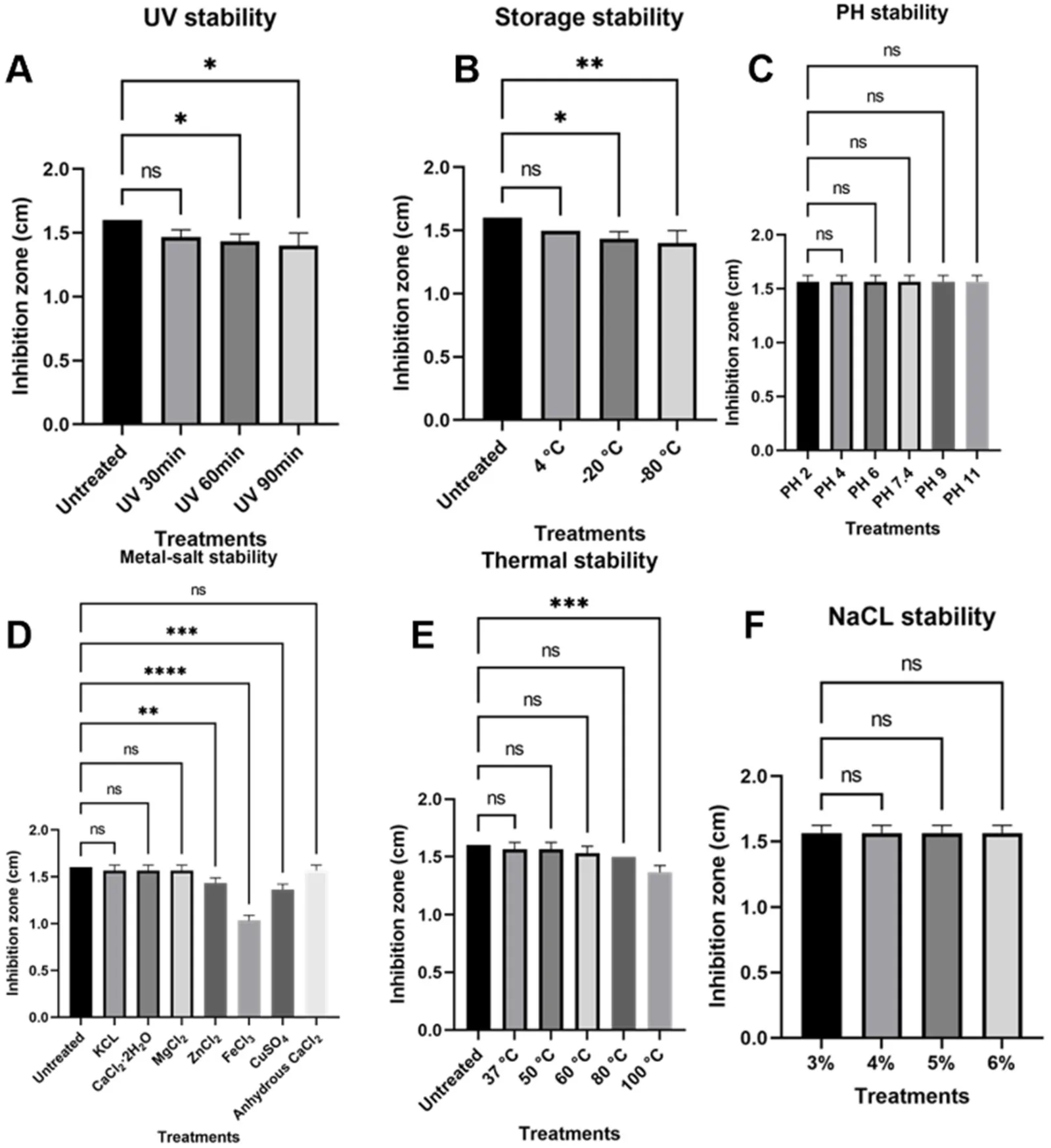
Functional physicochemical stability of DLP4. Antibacterial activity was measured after (**A**) ultraviolet exposure for 30, 60, and 90 min; (**B**) storage at 4, −20, and −80 °C; (**C**) incubation at pH 2, 4, 6, 7.4, 9, and 11; (**D**) exposure to KCl, CaCl_2_·2H_2_O, MgCl_2_, ZnCl_2_, FeCl_3_, CuSO_4_, and anhydrous CaCl_2_; (**E**) thermal treatment at 37, 50, 60, 80, and 100 °C; and (**F**) exposure to NaCl concentrations of 3–6%. Untreated DLP4 served as the control where applicable. Data are presented as mean ± SD of three independent replicates. Statistical comparisons are indicated by brackets. ns, not significant (*p* ≥ 0.05); * *p* < 0.05; ** *p* < 0.01; *** *p* < 0.001; and **** *p* < 0.0001.

DLP4 showed condition-dependent functional stability after physicochemical treatment (Figure 5). Untreated DLP4 produced a mean inhibition zone of 1.60 ± 0.00 cm and was defined as 100% activity. DLP4 showed relatively limited changes in inhibition-zone diameter following UV exposure, storage, and thermal treatment, with relative inhibition-zone values corresponding to approximately 85.4–97.9% of the untreated control. K^+^, Ca^2+^, and Mg^2+^ produced little change in measured inhibition zones, whereas Zn^2+^ and Cu^2+^ were associated with moderate reductions. The largest reduction in inhibition-zone diameter was observed in the presence of Fe^3+^, corresponding to approximately 64.6% of the untreated-control zone. DLP4 also produced inhibition zones comparable to the untreated control in the presence of 3–6% NaCl. Ion-only and sterile-water controls produced no inhibition zones, confirming that the observed antibacterial activity was due to DLP4. These results demonstrate retention of measurable antibacterial zone-forming activity under the tested conditions.

### 3.9. Phylogenetic Placement of DLP4

The precursor protein phylogeny placed *BSF* DLP4 within the broader insect defensin precursor group (Figure S3). In the recovered topology, DLP4 was positioned as a sister to the sampled dipteran defensin cluster rather than grouping with the hymenopteran defensin sequence, supporting classification as a defensin-like peptide within a dipteran associated lineage. Because the present analysis was based on a bootstrap supported neighbor joining framework rather than a model tested maximum likelihood approach, the topology is best interpreted as a supportive evolutionary context rather than as a fully resolved phylogenetic hypothesis.

### 3.10. Maturation and Physicochemical Properties

SignalP predicted that the 96-aa DLP4 precursor contains a classical Sec/SPI signal peptide with a cleavage site between residues 19–20, consistent with secretion. ProP predicted a dominant proprotein convertase cleavage at R56, defining a 40aa mature peptide enriched in cysteine and basic residues. A secondary predicted site at R89 was not adopted as the primary mature peptide boundary because it would truncate the cysteine framework. These results support DLP4 as a secreted defensin-like candidate generated from a larger precursor by signal peptide cleavage and downstream processing (Figure S4, Table 5). The predicted mature DLP4 peptide comprised 40 amino acids with a theoretical molecular weight of 4275.05 Da and a basic theoretical pI of 9.38. The sequence contained eight positively charged residues and two negatively charged residues, consistent with a strongly cationic AMP like profile. GRAVY was slightly negative, indicating a mildly hydrophilic overall character despite local hydrophobic segments, and the aliphatic index indicated moderate aliphatic content. ExPASy PeptideMass predicted an intact neutral mass of 4275.05 Da (average), providing a reference value for interpreting mass spectrometric data. These predicted properties are consistent with a defensin-like antibacterial candidate (Table 6).

### 3.11. Secondary-Structure and Antimicrobial Propensity Analyses

NetSurfP and related structure prediction outputs indicated a predominantly ordered peptide with a central α-helical segment and strand-like regions flanking a compact cysteine-rich core (Figure S5, Table 7). Predicted solvent accessibility indicated that most residues were exposed, whereas several central cysteine containing positions were relatively buried, consistent with a compact, cysteine stabilized peptide. Disorder probabilities were low across the sequence, indicating that mature DLP4 is predicted to be predominantly ordered rather than intrinsically disordered.

Both AMP-prediction tools classified mature DLP4 as an AMP with high confidence. AMP Scanner Vr2 returned a near-maximal AMP probability, and the CAMP predictor likewise classified DLP4 as an AMP across repeated runs. These in silico outputs are consistent with the peptide’s strongly cationic physicochemical profile and support AMP propensity, although classifier predictions still require experimental confirmation (Table 8).

### 3.12. Three-Dimensional Structural Modeling

**Figure 6:**
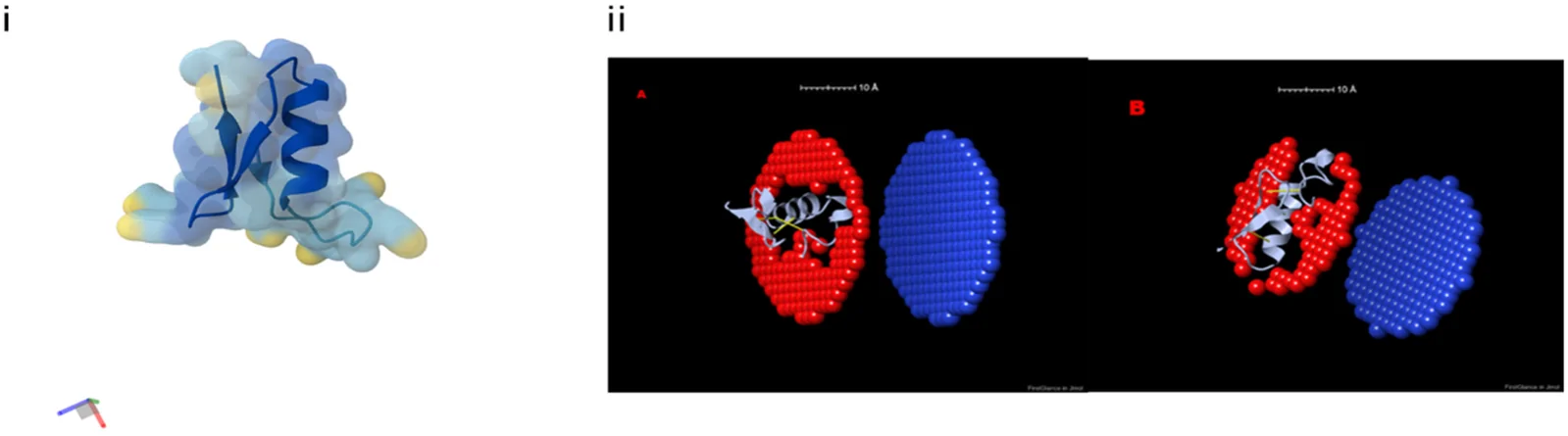
Predicted three-dimensional structure and membrane association of mature DLP4. (**i**) AlphaFold 3 predicted structure of mature DLP4 showing a compact globular fold with a central α-helix, β-strand elements, and connecting loops within a semi transparent molecular surface. (**ii**) PPM 3.0 predicted membrane placement of mature DLP4 in two membrane presets: (**A**) DOPC bilayer and (**B**) Gram-positive bacterial plasma membrane. Boundary markers indicate the hydrophobic core limits of the bilayer. The combined structural and membrane placement analyses support DLP4 as a compact, cysteine-rich, membrane-associated antibacterial peptide candidate.

ColabFold/AlphaFold2 generated top-ranked DLP4 models with mean pLDDT values in the low-to-mid 80s, with lower-confidence residues concentrated mainly at the termini. Comparison of the MSA-enabled and single-sequence runs revealed a broadly consistent predicted structural core, with some variability in terminal and loop regions. Given the short length and cysteine-rich composition of DLP4, loop geometry, disulfide connectivity, and atomic-level structural details should be considered provisional until experimentally determined. Overall, the models were consistent with a compact defensin-like fold and were used as supportive in silico evidence rather than as experimental determination of the DLP4 tertiary structure(Figure S6, Table 9). Structural modeling of mature DLP4 using AlphaFold Server generated a compact α/β fold consistent with a defensin-like peptide architecture (Figure 6i). The predicted model contained a prominent central α-helix together with adjacent β-strand elements connected by short loop regions, yielding a globular organization rather than an extended conformation. In the surface representation, the peptide appeared structurally compact, supporting the compatibility of the DLP4 sequence with an ordered tertiary fold. The predicted organization was compatible with structural features commonly associated with cysteine-rich defensin-like peptides. This prediction provides supportive structural context for DLP4 [63].

### 3.13. Predicted Membrane Association

PPM predicted mature DLP4 to favor peripheral membrane association in both tested membrane presets, indicating interfacial binding rather than transmembrane insertion. In both the DOPC bilayer and Gram-positive bacterial plasma membrane, DLP4 showed favorable predicted transfer energies, shallow insertion depth, and a high tilt angle relative to the membrane normal. Collectively, these outputs support a cautious membrane interface binding hypothesis typical of defensin-like AMPs, but they do not demonstrate membrane disruption or pore formation (Figure 6ii(A,B)).

### 3.14. Solvent-Dependent Secondary Structure of DLP4

**Figure 7:**
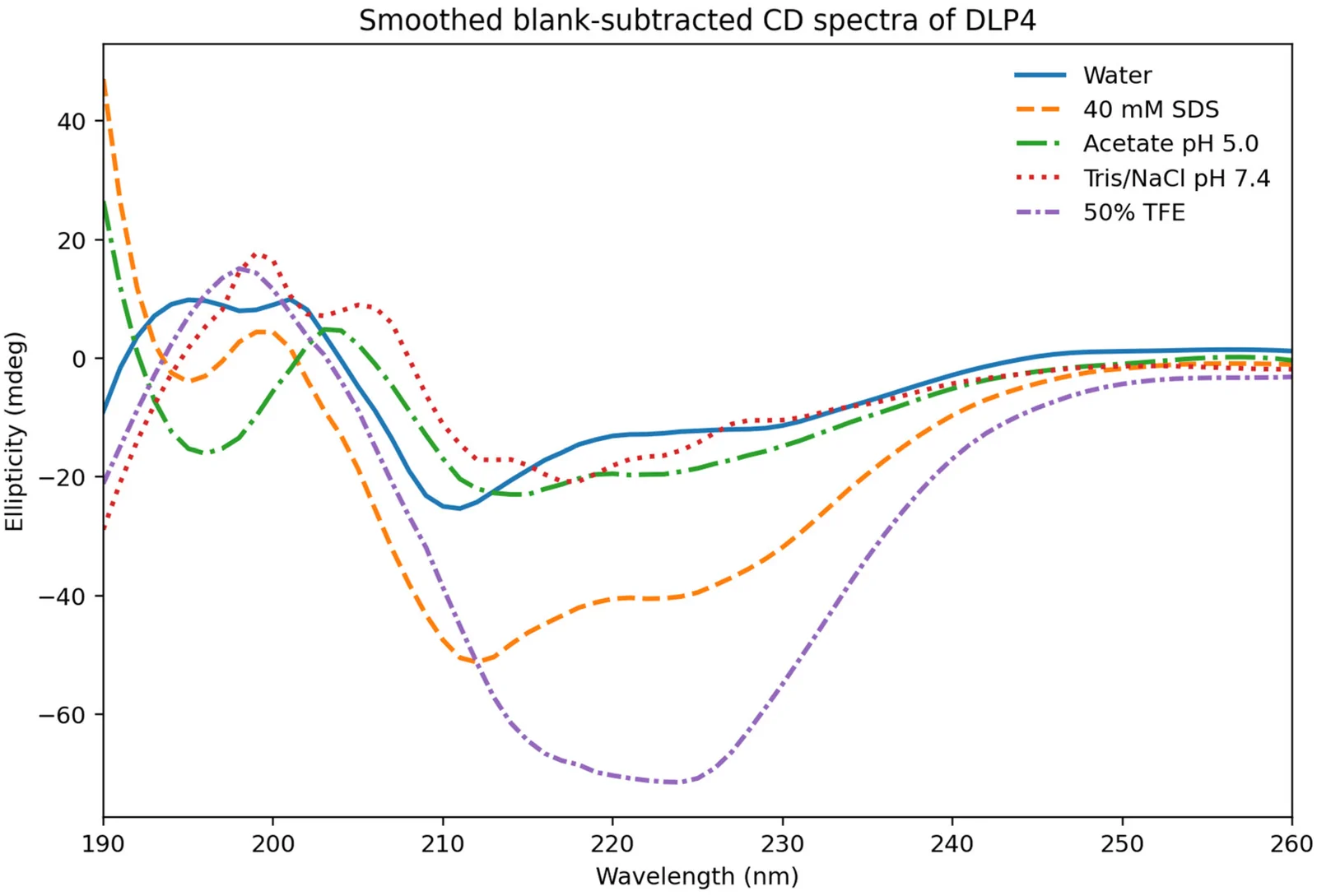
Smoothed blank subtracted far-UV circular dichroism spectra of DLP4 under five solvent/buffer conditions. Spectra are shown for DLP4 in water, 40 mM SDS, 5 mM sodium acetate + 15 mM NaCl (pH 5.0), 5 mM Tris-HCl + 150 mM NaCl (pH 7.4), and 50% TFE. DLP4 showed clear environment dependent conformational behavior, with the strongest ordered state profile observed in 50% TFE, followed by 40 mM SDS, whereas the Tris HCl/NaCl condition produced the weakest profile. The acetate condition showed an intermediate response between the aqueous baseline and the more structure promoting SDS and TFE environments.

Far-UV circular dichroism spectroscopy showed clear environment-dependent changes in the conformation of DLP4 (Figure 7). The spectra indicated a mixed structural state in water and increased helix-like ordering in SDS and particularly in TFE, whereas acetate and Tris/NaCl produced different intermediate spectral profiles. Exploratory deconvolution using the DS5-4 reference basis was performed to support comparison among conditions; however, several fits showed relatively high NRMSD values, particularly the Tris/NaCl condition. Therefore, the calculated secondary-structure fractions in Table 10 are presented as approximate estimates, and interpretation was based primarily on qualitative differences in spectral shape and relative conformational changes rather than on the exact percentage assigned to each secondary-structure class. Cross analysis with additional reference bases consistently supported increased helix-like ordering in SDS and TFE, whereas the estimates obtained for acetate and Tris/NaCl were more dependent on the selected reference basis.

## 4. Discussion

The present study integrated bacterial challenge-dependent hemolymph activity and antimicrobial-peptide transcription with direct functional and structural characterization of DLP4. Hemolymph antibacterial activity varied according to treatment and sampling time, while DLP4 and CLP1 showed distinct transcriptional responses at 24 h. Synthetic mature DLP4 exhibited strong but strain-dependent activity against Gram-positive bacteria, particularly *Bacillus subtilis* and *Listeria ivanovii*, whereas no MIC endpoint was detected against the tested *Escherichia coli* strains. DLP4 retained measurable antibacterial activity under a broad range of thermal, ultraviolet, storage, pH, NaCl, and metal-ion conditions. These results indicate substantial functional robustness under the tested assay conditions.

The significant treatment × time interactions observed in the hemolymph assays indicate that antibacterial activity was dynamic rather than constitutively elevated following injection or bacterial challenge. Such temporal variation is compatible with sequential processes involved in insect humoral immunity, including microbial recognition, immune-pathway activation, AMP transcription, precursor translation and processing, secretion, peptide degradation, and microbial clearance [22,24,39,68,69,70,71,72,73,74]. Black soldier fly larvae possess a broad repertoire of defensins, cecropins, attacins, diptericins, and other immune effectors whose expression can vary according to microbial exposure, diet, developmental stage, and tissue [22,69,73,74]. The present results therefore support the established view that BSF larvae can develop an inducible systemic antibacterial state.

Systematic insect AMP studies have shown that some peptides provide highly pathogen-specific protection, whereas others act through partly overlapping or cooperative mechanisms [22,69,72,73,75]. This distinction was evident when the hemolymph and synthetic-peptide results were compared. Challenged hemolymph inhibited both *S. aureus* and *E. coli*, whereas synthetic DLP4 produced no detectable MIC against the three tested *E. coli* strains. The anti-*E. coli* activity of the hemolymph must therefore involve other antibacterial components, either individually or in combination. Cecropin-like, attacin-like, and diptericin-like peptides are plausible contributors because related insect peptides frequently display stronger activity against Gram-negative bacteria [32,39,76,77,78]. The hemolymph assay consequently provides biological context for inducible antibacterial activity, whereas the peptide experiments define the intrinsic activity spectrum and physicochemical behaviour of DLP4.

DLP4 transcription increased following NaCl injection as well as bacterial challenge, demonstrating that the response was not specific to bacterial recognition. The significant induction following NaCl injection indicates that injection-associated tissue injury, vehicle administration, stress signalling, or other consequences of experimental manipulation contributed substantially to DLP4 regulation. Accordingly, the NaCl-injected group represents the appropriate procedural reference for distinguishing injection-associated responses from additional bacterial effects. Although the highest mean DLP4 expression was observed following *S. aureus* challenge, the present data do not establish a significant bacterial-specific increase relative to NaCl injection. DLP4 should therefore be interpreted as broadly responsive to injection-associated disturbance rather than as a strictly pathogen-specific transcriptional marker [22,48,68,69,73]. Nevertheless, the higher mean response following *S. aureus* challenge is biologically compatible with the strong activity of mature DLP4 against Gram-positive bacteria. In established insect immune models, lysine-type peptidoglycan from bacteria such as *S. aureus* is commonly associated with Toll-related immune activation, whereas diaminopimelic-acid-type peptidoglycan from many Gram-negative bacteria strongly activates Imd-associated responses [72]. The DLP4 pattern therefore supports its association with the early antibacterial response.

CLP1 showed a contrasting transcriptional pattern, with reduced expression following needle pricking, NaCl injection, and *S. aureus* challenge but increased expression after *E. coli* exposure. Cecropins are generally linear amphipathic peptides with comparatively strong activity against Gram-negative bacteria, and their transcription is frequently associated with Imd-related signalling in dipteran insects [24,69,72]. Increased CLP1 expression after *E. coli* challenge is therefore compatible with bacterial-species-dependent regulation. These findings provide biological justification for selecting DLP4 for direct functional characterization.

The MIC results provided direct evidence that mature DLP4 possesses strong antibacterial activity. *B. subtilis* KCCM 11316 was the most susceptible strain, with an MIC of 0.03–0.05 µM, followed by *L. ivanovii* ATCC 19119 at 0.1–0.2 µM. The examined *S. aureus* strains were inhibited at approximately 0.44–1.7 µM, while *S. hyicus* ACCC 61734 was inhibited at 0.9–1.1 µM. These results demonstrate substantial potency but also clear strain dependence. [15,20,79,80]. Differences among bacterial strains may arise from variation in surface charge, membrane phospholipid composition, peptidoglycan organization, teichoic-acid modification, extracellular polymers, proteolytic activity, and inducible peptide-resistance mechanisms [48,54,81,82].

The absence of detectable activity against the tested *E. coli* strains is consistent with the Gram-positive preference of many arthropod defensins. The outer membrane of Gram-negative bacteria provides an additional permeability barrier that can restrict peptide access to the cytoplasmic membrane. Lipopolysaccharide interactions, surface-charge modification, peptide sequestration, proteolysis, efflux, and envelope-stress responses may further reduce susceptibility [10,83]. From a food-science perspective, the selective spectrum of DLP4 is not necessarily a disadvantage. Targeted antibacterial activity may be useful in systems where Gram-positive pathogens or spoilage organisms are the primary concern. The strong activity against *L.*, *S.*, and *B.*-related bacteria provides a biological rationale for further evaluation in food systems in which these organisms are relevant.

Previous studies have already investigated the antibacterial mechanism of DLP4 and provide a useful framework for interpreting the present structural results. DLP4 has been reported to damage the bacterial cell wall and membrane, alter cell morphology, promote ion leakage, bind bacterial genomic DNA, disturb cell-cycle progression, and interfere with macromolecular synthesis. The extent of membrane disturbance reported here differed among bacterial strains and experimental conditions. Such variation may depend on peptide concentration, exposure time, growth phase, cell-envelope composition, and bacterial resistance mechanisms. The strain-dependent MIC values observed in the present study are consistent with this concept. Organisms that allow more efficient peptide binding, envelope disruption, or intracellular access would be expected to show lower MICs than strains possessing stronger surface or stress-response barriers.

LC–MS/MS analysis of the active F3 fraction revealed a mixed AMP profile, including the conserved ATCDLL motif shared among DLP1–DLP4, a distinct cysteine-rich defensin-like peptide, CLP2, and another cecropin-like AMP candidate. This composition is consistent with the antibacterial profile of F3, which inhibited both *S. aureus* and *E. coli* but showed greater activity against *S. aureus*. Defensin-like peptides are commonly associated with stronger activity against Gram-positive bacteria, whereas cecropin-like peptides frequently exhibit substantial activity against Gram-negative bacteria. Thus, the coexistence of defensin-like and cecropin-like components in F3 provides a plausible molecular basis for its activity against both bacterial classes. In particular, the detected cecropin-like components may have contributed to the inhibition of *E. coli*, whereas DLP-family/defensin-like peptides may have contributed more strongly to the activity against *S. aureus*. However, because F3 remained a heterogeneous peptide mixture and the individual components were not isolated and functionally tested separately, the antibacterial activity cannot be assigned definitively to any single peptide [53,59,84,85,86]. The computational structural and membrane-positioning results are consistent with a possible model in which DLP4 initially associates with the bacterial envelope and may subsequently access additional cellular targets. However, this represents a mechanistic hypothesis rather than an experimentally demonstrated pathway. PPM predicted shallow, peripheral membrane association rather than deep transmembrane insertion, but such positioning does not establish membrane binding, translocation, disruption, or pore formation.

The mature 40-residue DLP4 peptide had a theoretical molecular mass of approximately 4.28 kDa, a pI of 9.38, six cysteine residues, and eight positively charged residues. These characteristics are consistent with cationic insect defensins [61,62,63,87]. AlphaFold- and NetSurfP-based models supported a compact α/β-like organization, but these predictions should not be treated as proof of the atomic structure. The conformation of short cysteine-rich peptides depends strongly on disulfide connectivity, oxidation state, and solvent environment. The models are therefore most informative when interpreted together with the experimental MIC and circular-dichroism results.

Circular-dichroism analysis showed that DLP4 conformation was environment dependent. The peptide displayed a mixed structural profile in water but became more ordered in SDS and particularly in TFE. Increased ordering in an anionic membrane-mimetic environment may be biologically relevant because many AMPs undergo conformational stabilization after approaching negatively charged bacterial surfaces. Nevertheless, the CD results demonstrate environment-dependent folding rather than direct membrane disruption [43,65,66,67,81]. This agreement supports the reproducibility of DLP4 conformational responsiveness. Increased ordering in SDS may be biologically relevant because many AMPs become more structured after approaching negatively charged bacterial membranes.

DLP4 retained substantial measurable antibacterial activity following UV, storage, and thermal treatments and continued to produce clear inhibition zones under the evaluated pH, NaCl, and metal-ion conditions. Relative inhibition-zone diameters following UV, storage, and thermal treatments were approximately 85.4–97.9% of the untreated-control value, while inhibition zones in the presence of 3–6% NaCl were comparable to the untreated control. Among the tested metal ions, Fe^3+^ was associated with the greatest reduction in inhibition-zone diameter, whereas Zn^2+^ and Cu^2+^ produced smaller reductions. These findings indicate that DLP4 retained zone-forming antibacterial function under a range of physicochemical conditions. However, inhibition-zone diameter depends not only on antibacterial potency but also on peptide diffusion through agar. Changes in ionic strength, peptide charge, aggregation, and peptide–matrix interactions caused by salts or metal ions may therefore influence measured zone size independently of changes in intrinsic antibacterial potency. Accordingly, the relative inhibition-zone values should be interpreted as measure of functional activity under the assay conditions.

From a food-science perspective, the strong in vitro activity of synthetic DLP4 against several Gram-positive bacteria and its retention of measurable antibacterial activity under a range of physicochemical conditions provide a rationale for further evaluation in food systems. Its antibacterial spectrum suggests that future studies should particularly examine foodborne or spoilage organisms with Gram-positive cell envelopes rather than assume broad activity against mixed microbial populations. However, the present study did not evaluate DLP4 in actual food matrices or assess its behaviour during food processing, interactions with food components, sensory effects, bioavailability, or safety following food-related exposure or the disulfide bonding. Therefore, the present findings should be interpreted as establishing in vitro antibacterial potential rather than demonstrating suitability as a food preservative. Further matrix-based efficacy, toxicological, formulation, and sensory studies will be required before practical food applications can be considered.

Overall, this study links the inducible antibacterial response of *Hermetia illucens* with the functional properties of synthetic mature DLP4. Hemolymph activity and the distinct expression patterns of DLP4 and CLP1 indicate that the antibacterial response involves multiple immune effectors and cannot be attributed solely to DLP4. Synthetic DLP4 showed strong but strain-dependent activity, particularly against *Bacillus subtilis*, *Listeria ivanovii*, and several *Staphylococcus* strains, while no MIC endpoint was observed for the tested *Escherichia coli* strains. DLP4 also remained active after most thermal, ultraviolet, storage, pH, salt, and metal-ion treatments, although Fe^3+^ markedly reduced activity. These findings identify synthetic DLP4 as a Gram-positive-selective antimicrobial candidate that warrants further evaluation. Studies using representative food matrices and processing conditions, together with safety, formulation, sensory, and bioavailability assessments, are required before its suitability for food applications can be established.

## 5. Conclusions

This study integrated bacterial challenge, hemolymph antibacterial activity, gene-expression analysis, bioassay-guided fractionation, LC–MS/MS, and functional characterization to investigate the antimicrobial response of *Hermetia illucens* and the properties of mature DLP4. Bacterial challenge produced treatment- and time-dependent changes in hemolymph antibacterial activity, while DLP4 and CLP1 showed distinct transcriptional patterns, demonstrating that the BSF immune response involves multiple differently regulated antimicrobial effectors. Antibacterial activity was concentrated mainly in the early C18-SPE fractions, with F3 showing the highest activity. LC–MS/MS detection of a conserved DLP-family motif and a cysteine-rich defensin-like peptide provided molecular support for the presence of defensin-family components in this active fraction. The complete 40-amino-acid sequence of synthetic DLP4 was confirmed by LC–MS/MS. Synthetic DLP4 exhibited potent but strain-dependent antibacterial activity, with greater effectiveness against Gram-positive bacteria, including *Bacillus subtilis*, *Listeria ivanovii*, and several *Staphylococcus* strains. No MIC endpoint was detected against the tested *Escherichia coli* strains within the evaluated concentration range. DLP4 retained measurable antibacterial activity under a broad range of heat, ultraviolet, storage, pH, NaCl, and metal-ion conditions. Its cationic, cysteine-rich sequence and experimentally observed environment-dependent conformational behaviour, together with in silico predictions of a defensin-like fold and peripheral membrane association, provide complementary molecular context for its antibacterial activity. The computational predictions should be regarded as supportive hypotheses rather than experimental demonstration of tertiary structure or membrane-disrupting mechanism. Overall, DLP4 represents a physicochemically robust and predominantly Gram-positive-active antimicrobial candidate. Further studies should evaluate its safety, mechanism of action, formulation requirements, and effectiveness in real food matrices, where processing conditions may influence antimicrobial performance.

## Figures and Tables

**Table 1:** AMP gene primers.

Target	Forward Primer	Reverse Primer
DLP4 (defensin-like-peptide 4)	ACCAGTCGTTGCTGAAGTA	TCACAGGTTGCTCGTTTT
CLP1 (cecropin-like-peptide 1)	TCGTTGTTTTCGCTGTTG	CATTGGCTCCTTGCTGA
Actin	ACGAAGCACAAAGCAAGC	AGCCTCGGTCAACAGCA

**Table 2:** Stepwise RP-SPE elution scheme (F1–F10) showing ACN percentage and corresponding solvent volumes (ACN and 0.05% TFA water) per 20 mL fraction.

Fraction ID	ACN (%)	Acetonitrile (mL)	TFA Water (mL)
F1	10	2	18
F2	20	4	16
F3	30	6	14
F4	40	8	12
F5	50	10	10
F6	60	12	8
F7	70	14	6
F8	80	16	4
F9	90	18	2
F10	100	20	0

**Table 3:** LC–MS/MS identification of peptide sequences in the F3 fraction.

ID	Database Accession	Gene/Locus	Identified Sequence	Score	aa	Mass	*m*/*z*	z	RT (min)	Peak Area
**DLP-family peptide (DLP1–DLP4 shared motif)**	DLP4|DLP4_mature_*Hermetia_illucens*	DLP-family; multiple matching loci	ATC(+57.02)DLL	21.18	19	2024.956	692.32935	4	8.6615	374,000
**DLP-family peptide (DLP1–DLP4 shared motif)**	A0A7R8UB01; A0A7R8UC55	HERILL_LOCUS586; HERILL_LOCUS589	ATCDLLSPFGVGHAACAVHCIAMGRRGGWCDDRAVCNCR	15.07	39	4432.9486	634.28412	7	8.4333	135,000
**CLP2**	A0A7R8V1I5; A0A7R8Z0D4; A0A7R8YZ66	HERILL_LOCUS13354; HERILL_LOCUS13357; HERILL_LOCUS13360	GWWKRVFKPVEKLGQRVRDAGIQGLEIAQQGANVLATA	17.02	38	4189.3024	838.867	5	8.7758	1,060,000
**Cecropin-like AMP candidate**	A0A7R8YZ62		GWWKRVFKPVERLGQRVRDAGIQGLQIAQQGANVLATVRGGPPQQG	19.52	46	5021.744	1005.3537	5	8.8014	ND

**Table 4:** Strain-Dependent Antibacterial Activity of DLP4.

Bacterium and Strain	MIC (µM)
*S. aureus* KCCM 40881	0.61–1.11
*S. aureus* KCCM 12256	1.15–2.12
*B. subtilis* KCCM 11316	0.03–0.05
*S. aureus* ATCC 6538	0.44–0.93
*E. coli* KCCM 11234	Not detected within the tested range
*E. coli* CVCC 1515	Not detected within the tested range
*E. coli* ATCC 8739	Not detected within the tested range
*S. aureus* CICC 10001	1.4–1.7
*S. hyicus* ACCC 61734	0.9–1.1
*L. ivanovii* ATCC 19119	0.1–0.2

**Table 5:** SignalP and ProP outputs were used to define mature DLP4.

Tool (Version)	Input	Prediction	Position(s)/Score	Interpretation
SignalP 6.0	DLP4 precursor (96 aa)	Sec/SPI signal peptide	CS 19–20, Pr = 0.9834, SP(Sec/SPI) = 0.999817	Precursor is secreted, signal peptide defined as residues 1–19
ProP v1.0b	DLP4 precursor (96 aa)	Main convertase cleavage	R56, EHTREKR|AT, score = 0.931	Mature peptide defined as A57–K96 (40 aa) for downstream analyses
ProP v1.0b	DLP4 precursor (96 aa)	Secondary predicted cleavage	R89, GGWCDKR|AV, score = 0.788	Reported as secondary/internal site, not used to define primary mature peptide

**Table 6:** Physicochemical properties of mature DLP4 (A57–K96).

Property	Value	Interpretation
Length	40 aa	Mature peptide used for all downstream in silico analyses
Molecular weight (average)	4275.05 Da	Use for reporting peptide size (4.28 kDa)
Theoretical pI	9.38	Strongly basic/cationic peptide
Negatively charged residues (Asp + Glu)	2	Acidic residues are rare
Positively charged residues (Arg + Lys)	8	Cationic character, supports membrane-active AMP hypothesis
GRAVY	−0.130	Slightly hydrophilic overall (not strongly hydrophobic)
Aliphatic index	61.25	Moderate aliphatic content
Instability index	49.30	“Unstable” by ProtParam rule
Extinction coefficient (280 nm)	5875 M^−1^cm^−1^ (Cys as cystines)/5500 M^−1^cm^−1^ (Cys reduced)	Useful for UV-based concentration estimates if needed
Monoisotopic mass	4272.08 Da	neutral, reduced
[M+H]+	4273.0867 Da	monoisotopic, reduced
[M+H]+	4276.0575 Da	average, reduced

**Table 7:** Q3 secondary-structure segments of mature DLP4.

State (Q3)	Start	End	Length	State	Mean RSA	Fraction
C	1	1	1	Coil	0.6734634041786194	1
E	2	2	1	Strand	0.39211294054985046	1
C	3	3	1	Coil	0.14199355244636536	0
E	4	4	1	Strand	0.36088788509368896	1
C	5	12	8	Coil	0.4275641292333603	1
H	13	22	10	Helix	0.30806423388421533	0.5
C	23	26	4	Coil	0.5120752602815628	1
E	27	30	4	Strand	0.16317825391888618	0.25
C	31	34	4	Coil	0.4716319814324379	0.75
E	35	38	4	Strand	0.12092975433915854	0
C	39	40	2	Coil	0.6113950461149216	1

**Table 8:** The AMP Scanner and CAMP predictor returned AMP probabilities.

Model	Input	Class	Score	Notes
AMP Scanner Vr2 (Full model, February 2020)	Mature DLP4 (40 aa)	AMP	0.998–1.000	Exported summary and run output, high-confidence AMP call
CAMP predictor (run 1)	Mature DLP4 (40 aa)	AMP	0.99	AMP Probability
CAMP predictor (run 2)	Mature DLP4 (40 aa)	AMP	0.98	AMP Probability
CAMP predictor (run 3)	Mature DLP4 (40 aa)	AMP	0.98	AMP Probability

**Table 9:** ColabFold/AlphaFold2 structure for DLP4.

Run	MSA Sequences (A3M Headers)	Top Model (Ranked_0) Mean pLDDT	Top Model pTM	Top Model Max PAE	Residues with pLDDT < 70
ColabFold/AF2 with MSA (mmseqs2_uniref_env)	1265	81.89	0.54	22.09	1 2, 4–10
ColabFold/AF2 single sequence (no MSA)	1	86.15	0.56	21.45	1, 6–8
Between run comparison (ranked_0 vs. ranked_0)	-	-	-	-	Cα RMSD, 4.41 Å (all residues), 2.04 Å (residues 10–30)

**Table 10:** Secondary structure composition of DLP4 under different solvent conditions using DS5-4 CD deconvolution.

Condition	α-Helix-Like (%)	β-Structure (%)	Turn (%)	Other (%)	NRMSD
Water	20.6	23.7	38.7	17.0	0.187
40 mM SDS	38.3	1.5	28.6	31.6	0.415
Acetate, pH 5.0	18.4	17.7	36.1	27.9	0.405
Tris/NaCl, pH 7.4	31.4	0.0	24.7	43.9	0.793
50% TFE	55.2	0.0	44.8	0.0	0.493

## Data Availability

The original contributions presented in this study are included in the article/Supplementary Material. Further inquiries can be directed to the corresponding author.

## Supplementary Materials

The following supporting information can be downloaded at: https://www.mdpi.com/article/10.3390/foods15173107/s1, Table S1: Estimated viable bacterial dose per larva based on OD600-defined *S. aureus* and *E. coli* challenge suspensions; Table S2: Stability of Actin Ct values across control and bacterial-challenge treatments used for RT-qPCR normalization; Table S3. Distinct LC–MS/MS peptide-spectrum matches detected in the F3 fraction; Table S4. LC–MS/MS identification of synthetic DLP4 and its sequence-derived ions; Figure S1: Representative zone of inhibition assay against *Staphylococcus aureus* using hemo-lymph collected from BSF larvae subjected to control, procedural control, and bacterial challenge treatments. Sample labels are defined as follows, (a–c), untreated control (D1–D3), (d–f), needle-pierced only (D1–D3), (g–i), NaCl injection (D1–D3), (j–l), *Staphylococcus aureus* OD = 1 (D1–D3), (m–o), *S. aureus* OD = 2 (D1–D3), (p–r), *S. aureus* OD = 2.5 (D1–D3), (s–u), *Escherichia coli* OD = 1 (D1–D3), (v–x), *E. coli* OD = 2 (D1–D3), (y–za), *E. coli* OD = 2.5 (D1–D3). D1, D2, and D3 indicate day 1, day 2, and day 3 post-treatment, respectively. Figure S2: Representative zone of inhibition assay against *Escherichia coli* using hemolymph col-lected from BSFL subjected to control, procedural control, and bacterial challenge treatments. Sample labels are defined as follows, (A–C), untreated control (D1–D3), (D–F), needle-pierced only (D1–D3), (G–I), NaCl injection (D1–D3), (J–L), *Staphylococcus aureus* OD = 1 (D1–D3), (M–O), *S. aureus* OD = 2 (D1–D3), (P–R), *S. aureus* OD = 2.5 (D1–D3), (S–U), *Escherichia coli* OD = 1 (D1–D3), (V–X), *E. coli* OD = 2 (D1–D3), (Y–Za), *E. coli* OD = 2.5 (D1–D3). D1, D2, and D3 indicate day 1, day 2, and day 3 post-treatment, respectively. Figure S3: Bootstrap-supported precursor-protein phylogeny of BSF DLP4 and representative insect defensin or defensin-like sequences. The tree was constructed using accession-backed amino-acid precursor sequences retrieved from NCBI GenBank, including the translated 96-aa DLP4 precursor from KF805350.1. Sequences were aligned using a center-star progressive strategy with BLOSUM62 scoring, gap-rich columns were trimmed, and phylogeny was inferred by the neighbor-joining method from Poisson-corrected amino-acid distances. Branch support values are based on 1000 bootstrap pseudoreplicates. Dermacentor variabilis defensin 2 (AY159879.1) was used as the outgroup. The topology places BSF DLP4 within the broader insect defensin precursor group as sister to the sam-pled dipteran defensin cluster. Figure S4: DLP4 precursor architecture and predicted processing. SignalP predicted a Sec/SPI signal peptide with a cleavage site between residues 19–20 (Pr = 0.9834, SP probability = 0.999817). ProP predicted a dominant proprotein convertase cleavage at R56 (EHTREKR|AT, score = 0.931), defining the mature peptide as A57–K96 (40 aa). A secondary predicted site at R89 (GGWCDKR|AV, score = 0.788) is shown as a dashed marker and was not used to define the primary mature boundary. Figure S5: Predicted structural features and surface exposure of mature DLP4. NetSurfP predicted that mature DLP4 adopts a mixed secondary structure comprising approximately 50% coil, 25% helix, and 25% β-strand. Figure S6: Predicted 3D structure of mature DLP4 from ColabFold/AlphaFold2 (MSA-enabled run). The top-ranked model (ranked_0) is shown as a Cα trace colored by pLDDT (confidence). Figure S7: LC-MS/MS chromatogram of F3 fraction. Figure S8: LC-MS/MS chromatogram of synthetic DLP4. Figure S9: HPLC report of synthetic DLP4. Figure S10: ESI-MS report of the synthetic DLP4.
