# Supplementary material for: Molecular and Functional Characterization of Black Soldier Fly Derived Peptide Against Food-Relevant Bacteria
Source: Foods. 2026 Sep 1;15(17):3107. doi: 10.3390/foods15173107 (PMC13564798; doi:10.3390/foods15173107)
Supplement: Supplementary file 1 [file foods-15-03107-s001.zip › Figure S9.pdf]

HPLC REPORT

Column : 4.6×250mm,Boston Green C4 300A

Solvent A : 0.1% trifluoroacetic in 100% acetonitrile

Solvent B : 0.1% trifluoroacetic in 100% water

Gradient :

	A	B
0.01min	10%	90%
25min	60%	40%
25.1min	100%	0%
30min	STOP	

Flow rate : 1.0 mL/min

Wavelength : 220nm

Volume : 5ul

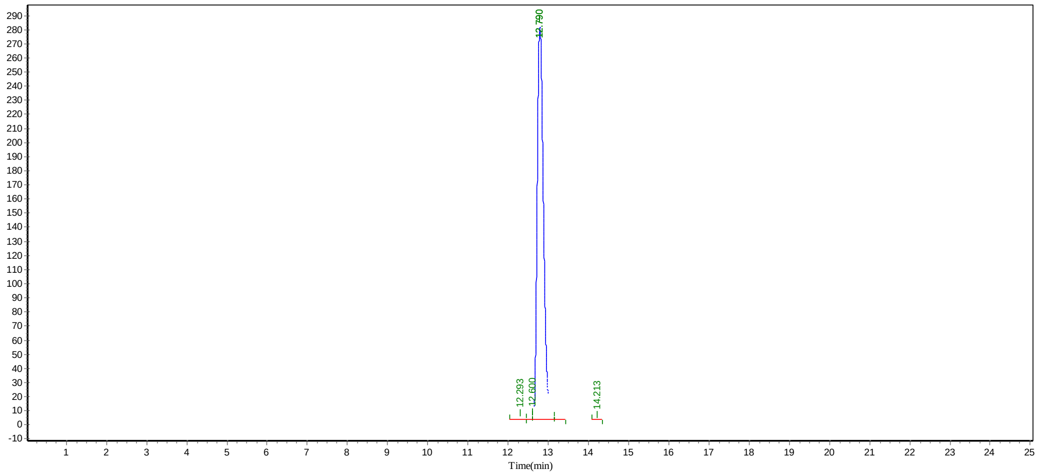

Peak No.	Ret Time	Height	Area	Conc..
1	12.293	1710.766	23062.502	0.7518
2	12.600	2375.751	13729.789	0.4476
3	12.790	280682.094	3016713.000	98.3358
4	12.790	1557.285	9391.401	0.3061
5	14.213	569.906	4869.450	0.1587
Total				100.0000
