# Supplementary material for: Molecular and Functional Characterization of Black Soldier Fly Derived Peptide Against Food-Relevant Bacteria
Source: Foods. 2026 Sep 1;15(17):3107. doi: 10.3390/foods15173107 (PMC13564798; doi:10.3390/foods15173107)
Supplement: Supplementary file 1 [file foods-15-03107-s001.zip › suplementary Figure S10.pdf]

MASS SPECTROMETRY REPORT

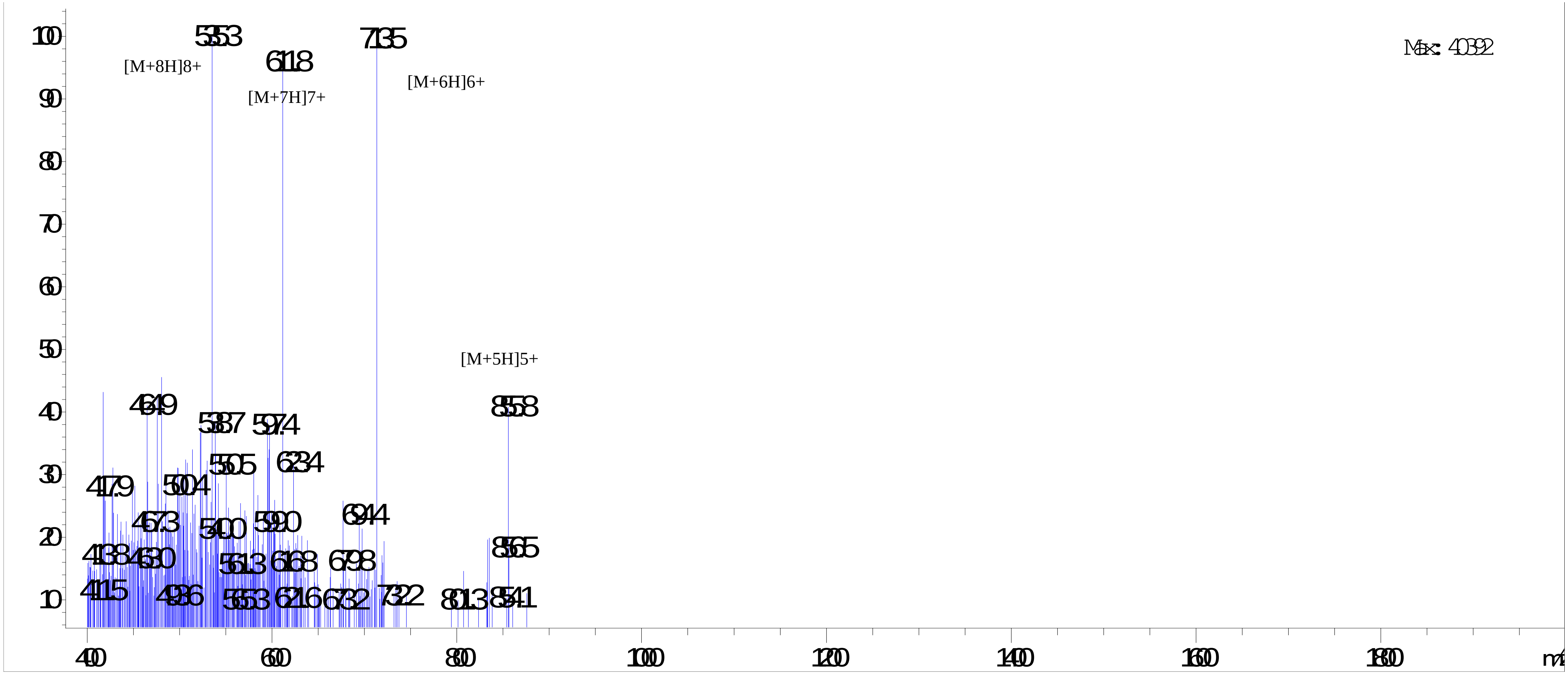

Sample Description		Instrument	Agilent-6125B		
Analyzed date:	2026-03-12	Probe:	ESI	Probe Bias:	＋4.5kv
		Nebulizer Gas Flow:	1.5L/min	Detector:	1.5kv
		CDL:	-20.0v	T. Flow:	0.2ml/min
M.W.:	4275.08	CDL Temp.:	250 ^{o}C	B. Conc.:	50%H2O/50%ACN
		Block Temp.:	200 ^{o}C		
