# Supplementary material for: Molecular and Functional Characterization of Black Soldier Fly Derived Peptide Against Food-Relevant Bacteria
Source: Foods. 2026 Sep 1;15(17):3107. doi: 10.3390/foods15173107 (PMC13564798; doi:10.3390/foods15173107)
Supplement: Supplementary file 1 [file foods-15-03107-s001.zip › supplementary material.pdf]

Table S1 Estimated viable bacterial dose per larva based on OD_{600}-defined *S.aureus* and *E.coli* challenge suspensions

Species	OD_{600}	Estimated CFU/mL	Injection volume	Estimated CFU/larva
S. aureus ATCC 6538	1.0	5.07×10^8	5 µL / 0.005 mL	2.53×10^6
S. aureus ATCC 6538	2.0	1.01×10^9	5 µL / 0.005 mL	5.07×10^6
S. aureus ATCC 6538	2.5	1.27×10^9	5 µL / 0.005 mL	6.33×10^6
S. aureus ATCC 6538	3.0	1.52×10^9	5 µL / 0.005 mL	7.60×10^6
E. coli ATCC 8739	1.0	2.75×10^8	5 µL / 0.005 mL	1.38×10^6
E. coli ATCC 8739	2.0	5.50×10^8	5 µL / 0.005 mL	2.75×10^6
E. coli ATCC 8739	2.5	6.88×10^8	5 µL / 0.005 mL	3.44×10^6
E. coli ATCC 8739	3.0	8.26×10^8	5 µL / 0.005 mL	4.13×10^6

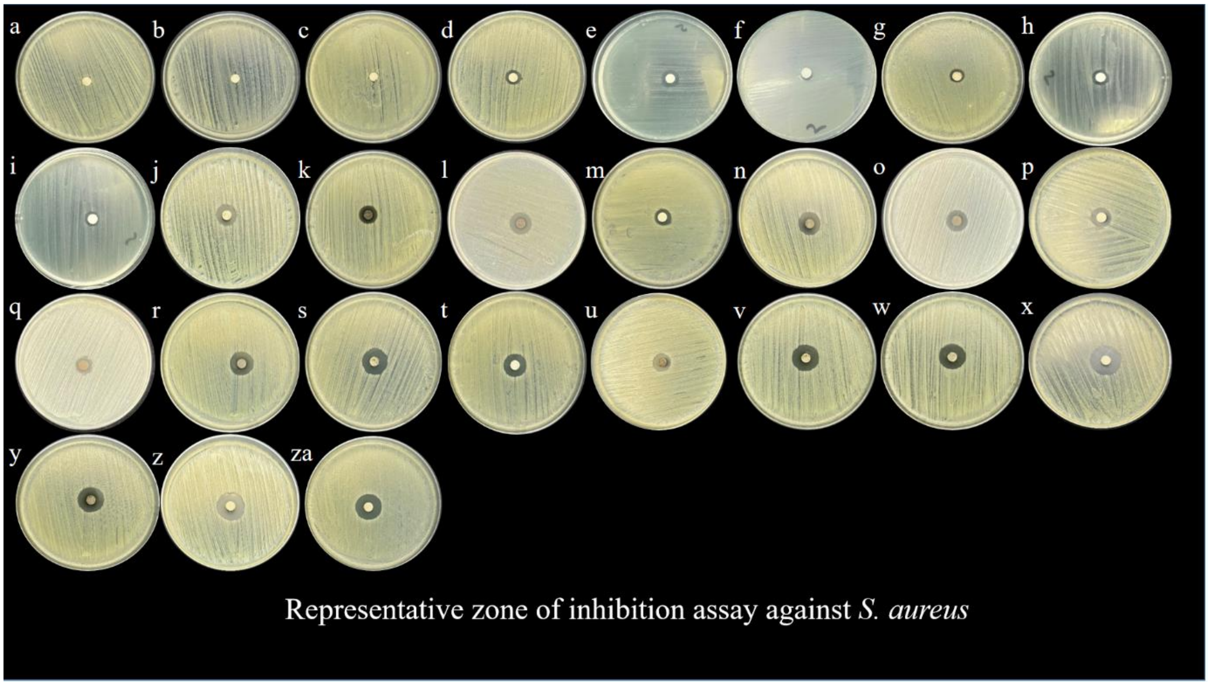

Figure S1. Representative zone of inhibition assay against *Staphylococcus aureus* using hemolymph collected from BSF larvae subjected to control, procedural control, and bacterial challenge treatments. Sample labels are defined as follows, a–c, untreated control (D1–D3), d–f, needle-pierced only (D1–D3), g–i, NaCl injection (D1–D3), j–l, *Staphylococcus aureus* OD = 1 (D1–D3), m–o, S. aureus OD = 2 (D1–D3), p–r, S. aureus OD = 2.5 (D1–D3), s–u, *Escherichia coli* OD = 1 (D1–D3), v–x, *E. coli* OD = 2 (D1–D3), y–za, *E. coli* OD = 2.5 (D1–D3). D1, D2, and D3 indicate day 1, day 2, and day 3 post-treatment, respectively.

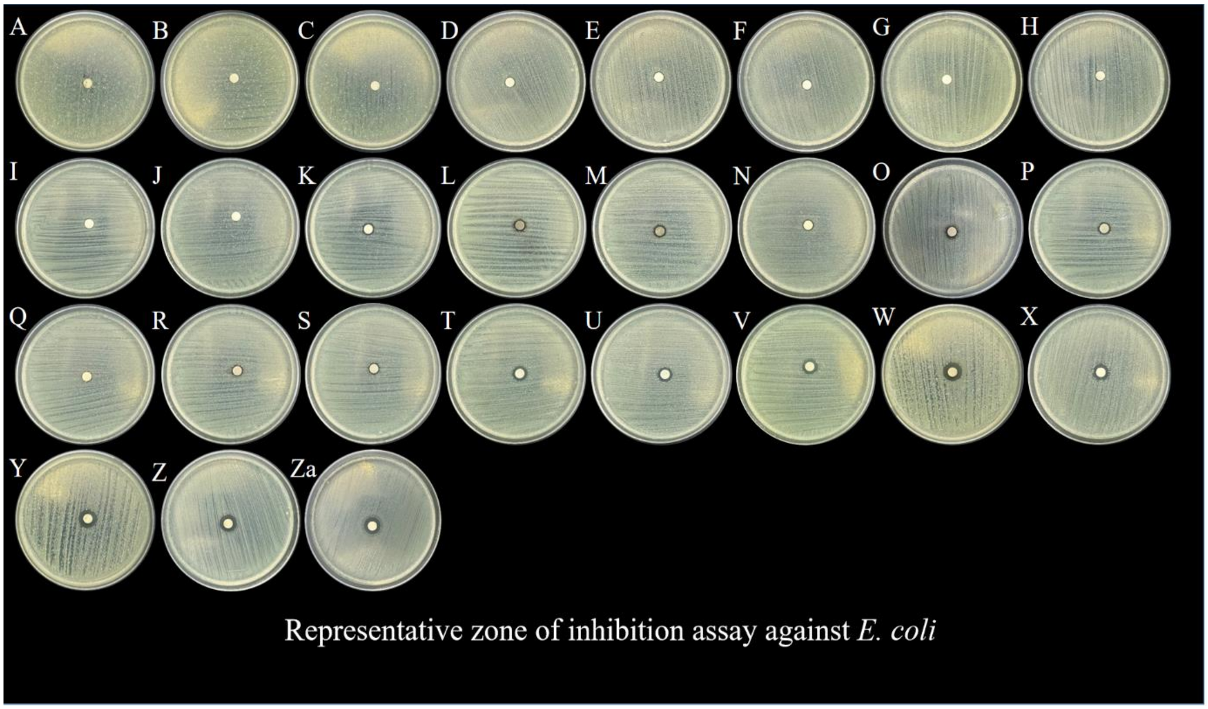

Figure S2. Representative zone of inhibition assay against *Escherichia coli* using hemolymph collected from BSFL subjected to control, procedural control, and bacterial challenge treatments. Sample labels are defined as follows, A–C, untreated control (D1–D3), D–F, needle-pierced only (D1–D3), G–I, NaCl injection (D1–D3), J–L, *Staphylococcus aureus* OD = 1 (D1–D3), M–O, S. aureus OD = 2 (D1–D3), P–R, S. aureus OD = 2.5 (D1–D3), S–U, *Escherichia coli* OD = 1 (D1–D3), V–X, *E. coli* OD = 2 (D1–D3), Y–Za, *E. coli* OD = 2.5 (D1–D3). D1, D2, and D3 indicate day 1, day 2, and day 3 post-treatment, respectively.

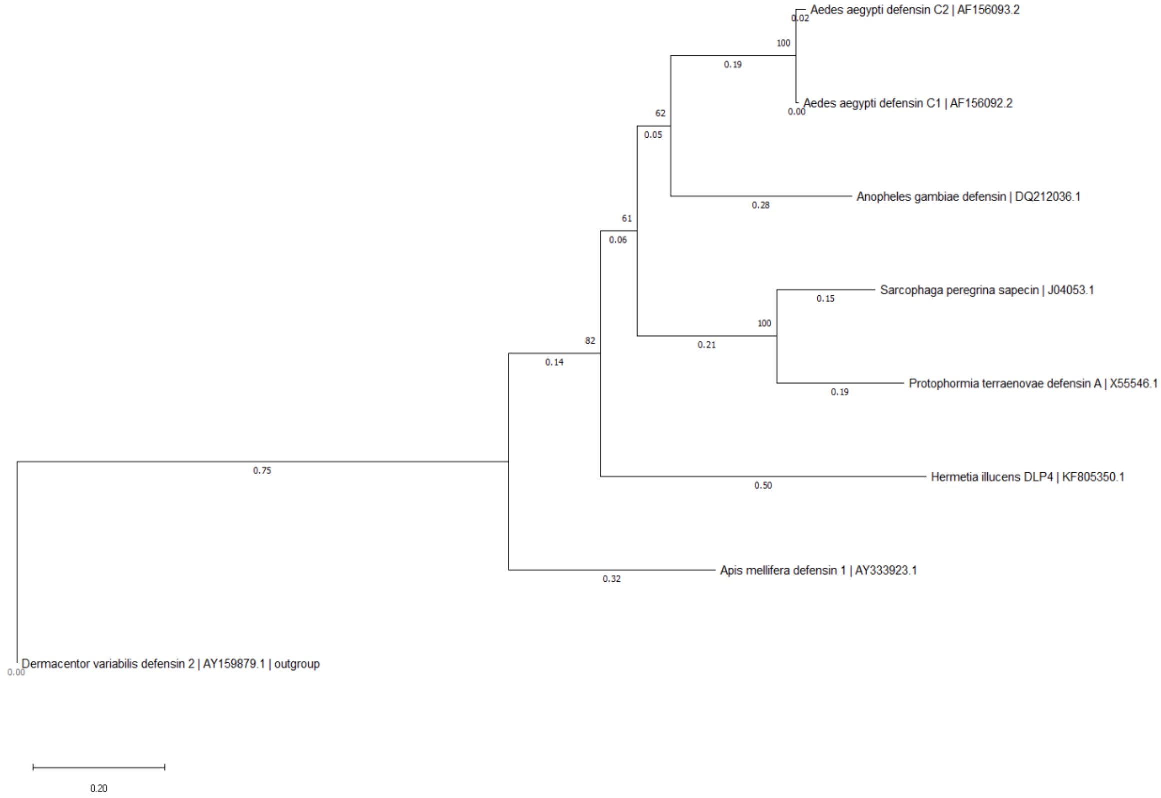

Figure S3. Bootstrap-supported precursor-protein phylogeny of *BSF* DLP4 and representative insect defensin or defensin-like sequences. The tree was constructed using accession-backed amino-acid precursor sequences retrieved from NCBI GenBank, including the translated 96-aa DLP4 precursor from KF805350.1. Sequences were aligned using a center-star progressive strategy with BLOSUM62 scoring, gap-rich columns were trimmed, and phylogeny was inferred by the neighbor-joining method from Poisson-corrected amino-acid distances. Branch support values are based on 1000 bootstrap pseudoreplicates. Dermacentor variabilis defensin 2 (AY159879.1) was used as the outgroup. The topology places *BSF* DLP4 within the broader insect defensin precursor group as sister to the sampled dipteran defensin cluster.

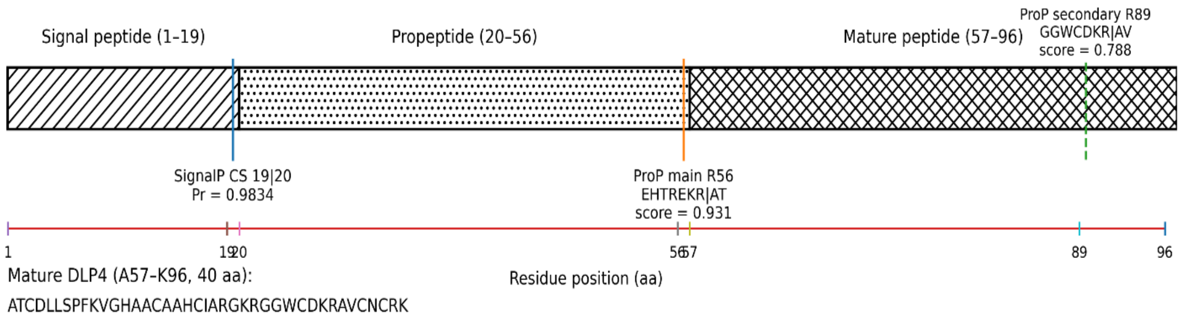

Figure S4. DLP4 precursor architecture and predicted processing. SignalP predicted a Sec/SPI signal peptide with a cleavage site between residues 19–20 (Pr = 0.9834, SP probability = 0.999817). ProP predicted a dominant proprotein convertase cleavage at R56 (EHTREKR|AT, score = 0.931), defining the mature peptide as A57–K96 (40 aa). A secondary predicted site at R89 (GGWCDKR|AV, score = 0.788) is shown as a dashed marker and was not used to define the primary mature boundary.

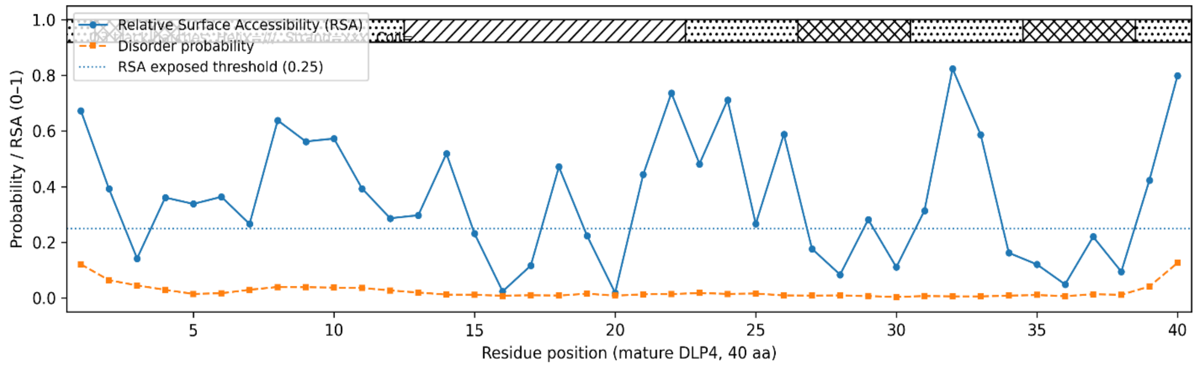

Figure S5. Predicted structural features and surface exposure of mature DLP4. NetSurfP predicted that mature DLP4 adopts a mixed secondary structure comprising approximately 50% coil, 25% helix, and 25% β-strand.

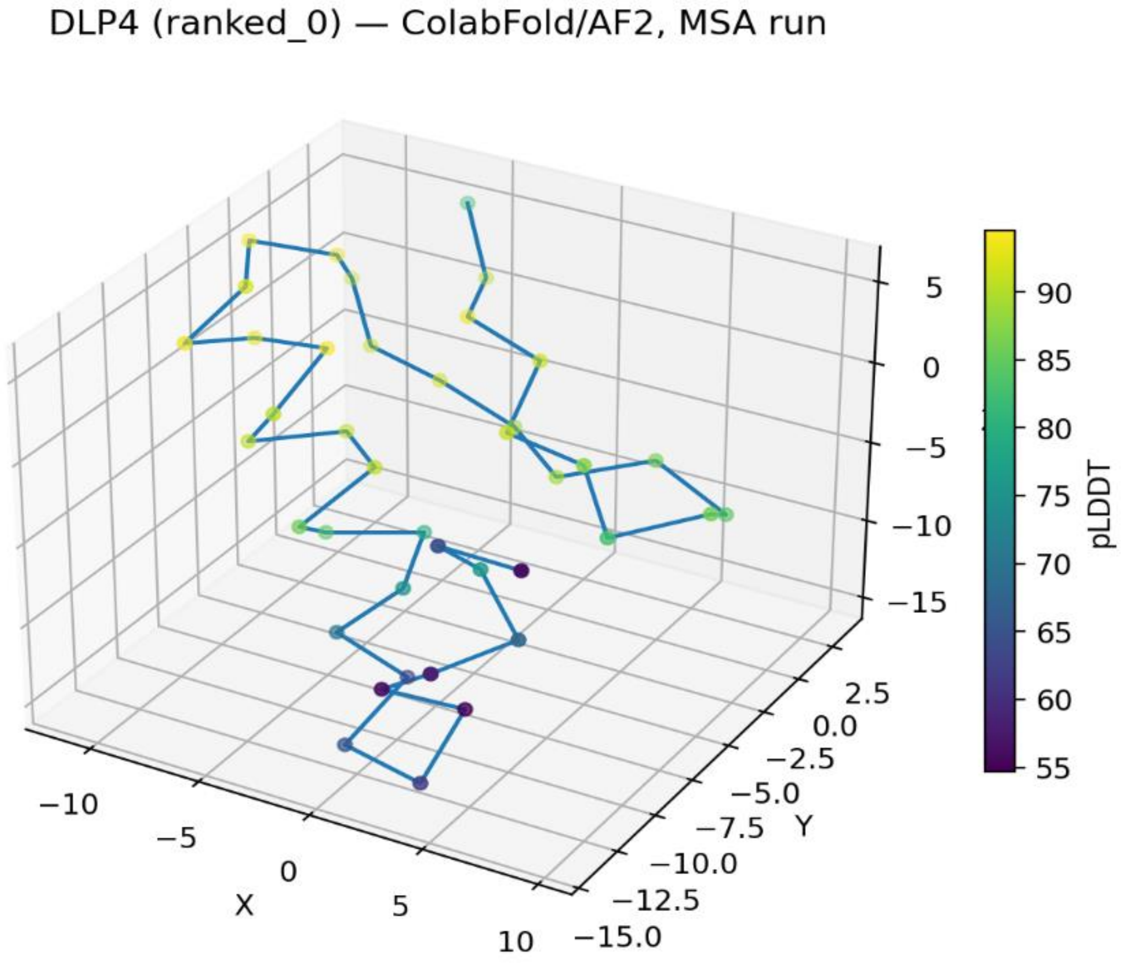

Figure S6. Predicted 3D structure of mature DLP4 from ColabFold/AlphaFold2 (MSA-enabled run). The top-ranked model (ranked_0) is shown as a Cα trace colored by pLDDT (confidence).

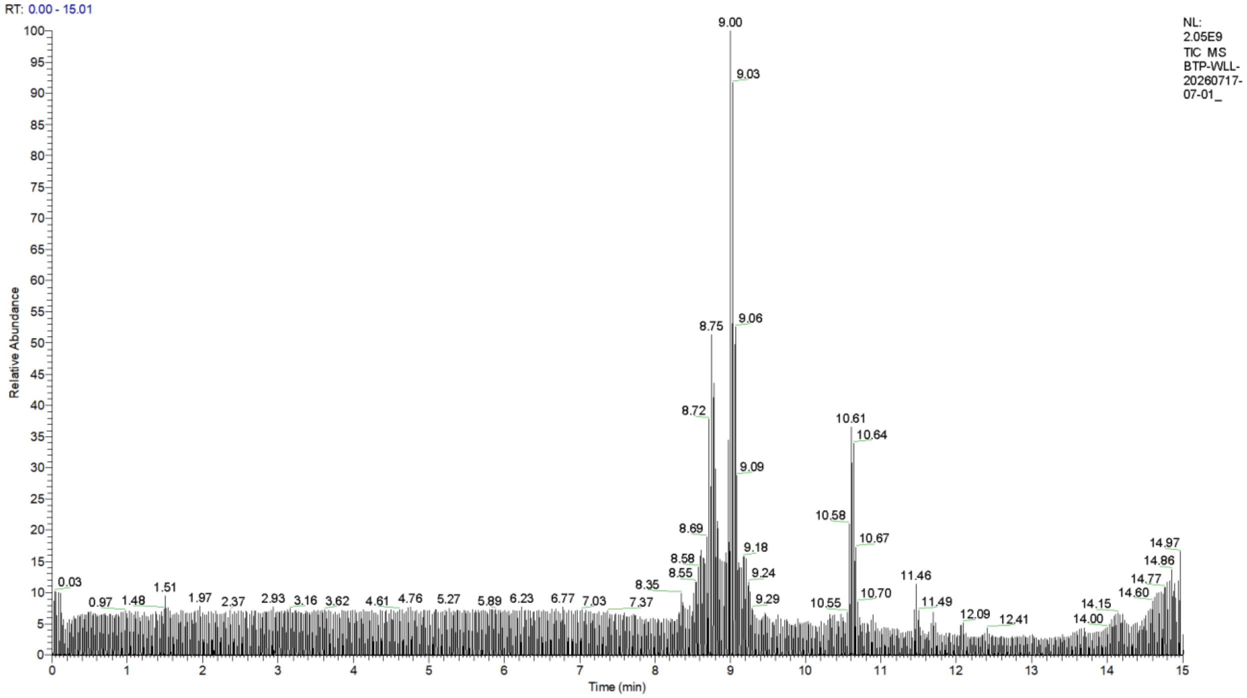

Figure S7. LC-MS/MS chromatogram of F3 fraction.

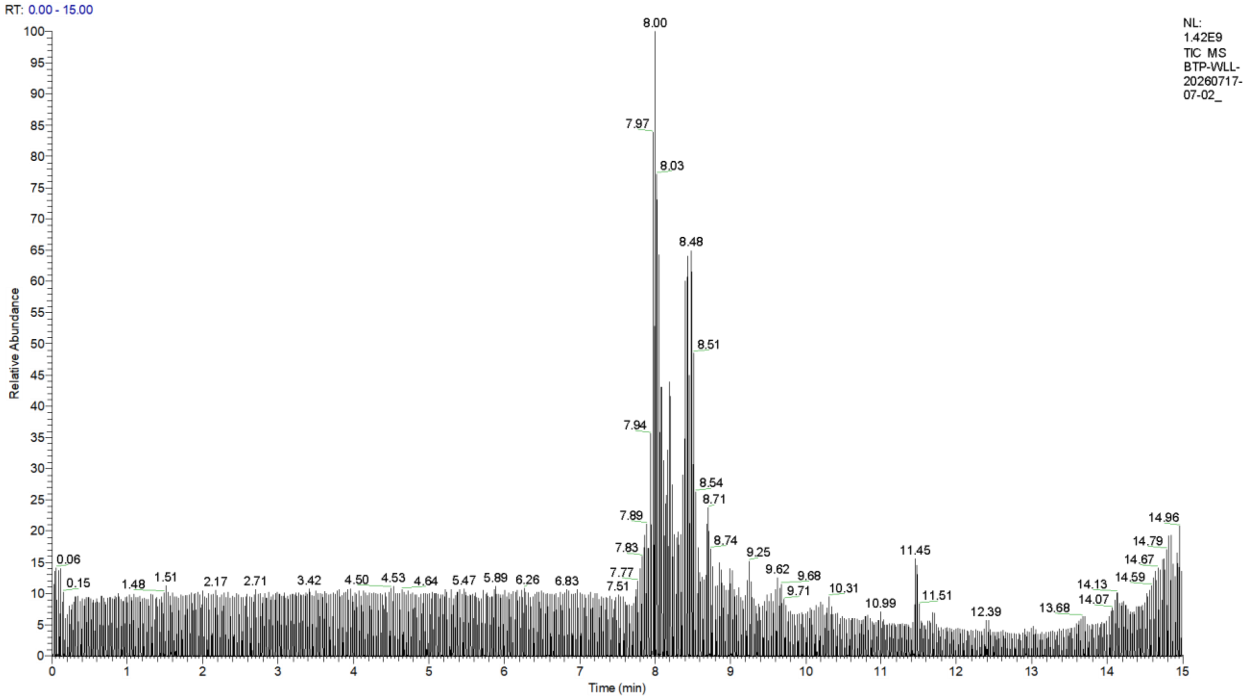

Figure S8. LC-MS/MS chromatogram of synthetic DLP4.

Table S2 Stability of Actin Ct values across control and bacterial-challenge treatments used for RT-qPCR normalization.

Treatment	Actin Ct 1	Actin Ct 2	Actin Ct 3	Mean ± SD
Control	19.150	19.301	19.428	**19.29 ± 0.14**
Needle-pricked	19.286	18.066	19.172	**18.84 ± 0.67**
NaCl	19.211	19.112	19.176	**19.17 ± 0.05**
S. aureus	19.348	19.696	18.447	**19.16 ± 0.64**
E. coli	19.688	19.690	19.192	**19.52 ± 0.29**

Values represent three independent biological replicates per treatment. No significant difference in Actin Ct values was detected among treatments (one-way ANOVA, $F_{4,10}$ = 0.94, p = 0.479).
